# Efficient CRISPR/Cas-Mediated Targeted Mutagenesis in Spring and Winter Wheat Varieties

**DOI:** 10.3390/plants10071481

**Published:** 2021-07-19

**Authors:** Florian Hahn, Laura Sanjurjo Loures, Caroline A. Sparks, Kostya Kanyuka, Vladimir Nekrasov

**Affiliations:** 1 Plant Sciences Department, Rothamsted Research, Harpenden AL5 2JQ, UK; florian.hahn@plants.ox.ac.uk (F.H.); laura.sanjurjo-loures@rothamsted.ac.uk (L.S.L.); caroline.sparks@rothamsted.ac.uk (C.A.S.); 2 Department of Biointeractions and Crop Protection, Rothamsted Research, Harpenden AL5 2JQ, UK; kostya.kanyuka@rothamsted.ac.uk

**Keywords:** CRISPR, Cas9, plant, genome editing, BAK1, eIF4E, wheat

## Abstract

CRISPR/Cas technology has recently become the molecular tool of choice for gene function studies in plants as well as crop improvement. Wheat is a globally important staple crop with a well annotated genome and there is plenty of scope for improving its agriculturally important traits using genome editing technologies, such as CRISPR/Cas. As part of this study we targeted three different genes in hexaploid wheat *Triticum aestivum*: *TaBAK1-2* in the spring cultivar Cadenza as well as *Ta-eIF4E* and *Ta-eIF(iso)4E* in winter cultivars Cezanne, Goncourt and Prevert. Primary transgenic lines carrying CRISPR/Cas-induced indels were successfully generated for all targeted genes. While BAK1 is an important regulator of plant immunity and development, Ta-eIF4E and Ta-eIF(iso)4E act as susceptibility (S) factors required for plant viruses from the *Potyviridae* family to complete their life cycle. We anticipate the resultant homozygous *tabak1-2* mutant lines will facilitate studies on the involvement of BAK1 in immune responses in wheat, while *ta-eif4e* and *ta-eif(iso)4e* mutant lines have the potential to become a source of resistance to wheat spindle streak mosaic virus (WSSMV) and wheat yellow mosaic virus (WYMV), both of which are important pathogens of wheat. As winter wheat varieties are generally less amenable to genetic transformation, the successful experimental methodology for transformation and genome editing in winter wheat presented in this study will be of interest to the research community working with this crop.

## 1. Introduction

Common wheat (*Triticum aestivum*) is one of the most important staple food crops in the world. The challenges that global agriculture currently faces, such as growth of the world’s population and climate change, dictate demand for technologies with a potential to accelerate crop breeding [1]. During the last decade, genome editing emerged as a powerful new breeding technique (NBT) [2] that enables targeted changes in crop genomes.

CRISPR/Cas is by far the most common plant genome editing technology nowadays due to its precision, versatility and ease of use [3]. It is an excellent tool for gene function studies as well as improvement of agriculturally important crop traits. In wheat, the CRISPR/Cas technology has been successfully used for both above-mentioned applications. For instance, traits such as disease resistance, yield, phosphorus-use efficiency, starch quality and herbicide tolerance are among those successfully improved using genome editing in this crop (for a recent comprehensive review on the topic, please refer to Li et al. [4]). In the vast majority of cases, wheat trait improvement by genome editing has been achieved via knocking out genes associated with the traits. Apart from gene knockouts, other genome editing applications, such as base editing, prime editing and those relying on homology-directed repair (HDR) have not been widely adopted in wheat due to the low efficiencies and requirements for further optimisation [4]. As bread wheat is an allohexaploid, it is important to have an efficient CRISPR/Cas setup as, in the majority of cases, for each particular gene, one needs to target six copies i.e., two per each of the three subgenomes (A, B and D).

As part of this study, we used CRISPR/Cas in a reverse genetics approach to target the *TaBAK1-2* gene, a homologue of the Arabidopsis *BAK1* gene encoding the BRI1-associated receptor kinase 1 (BAK1)—an important regulator of plant immunity and development [5,6], in the spring wheat cultivar Cadenza. Here we successfully knocked out all three *TaBAK1-2* homoeologues in primary transgenic lines and demonstrated transmission of the CRISPR/Cas-induced mutant alleles to the next generation (T1). We anticipate the resultant homozygous mutant lines will facilitate studies on the involvement of BAK1 in immune responses in wheat.

In the second part of the study, we tested the potential of the CRISPR/Cas system in wheat for generating resistance to bymoviruses in the family *Potyviridae*, some of which are serious pathogens of crops. For instance, wheat spindle streak mosaic virus (WSSMV) can pose a serious threat to wheat production in Europe and North America, while wheat yellow mosaic virus (WYMV)—in East Asia [7]. Here, we targeted *Ta-eIF4E* and *Ta-eIF(iso)4E* genes encoding highly conserved translation-initiation factors eIF4E and eIF(iso)4E, respectively, which serve as susceptibility (S) factors required for plant viruses from the *Potyviridae* family to complete their life cycle [8]. An analogous genome-editing-based strategy has already been successfully used in Arabidopsis, cucumber and cassava [9,10,11,12]. In addition, in barley, the conventional breeding strategies for generating resistance to bymoviruses barley mild mosaic virus (BaMMV) and barley yellow mosaic virus (BaYMV) are based on introducing recessive mutant alleles of the *eIF4E* gene [7,13]. In this study, we generated genome-edited wheat lines carrying indels in all three homoeologues of either *Ta-eIF4E* or *Ta-eIF(iso)4E*. These lines will be assessed for enhanced resistance to WSSMV in the follow-up study.

## 2. Materials and Methods

### 2.1. Target Sites

The single guide RNA (sgRNA) target sites were chosen using the CRISPOR online tool [14] or the Geneious software. The target genes were sequenced in all wheat varieties used for transformation to ensure the presence of the chosen sgRNA target sites (Table 1) in each subgenome of every chosen variety.

### 2.2. Plasmid Construction

#### 2.2.1. *TaBAK1-2*

Three guides (guides 1, 2 and 3; Figures S1–S3) targeting *TaBAK1-2* were delivered on separate constructs. Each guide was placed under the rice U6 promoter by cloning into the pUC19_rice_sgRNA_v2 vector (kindly provided by Keith Edwards, University of Bristol, Bristol, UK) using BtgZI, as previously described for pENTR4-sgRNA4 [15]. All three sgRNA plasmids were co-delivered along with pCas9-GFP [16] encoding the wheat codon-optimised Cas9, and pRRes1.111 [17] encoding the *bar* selectable marker into immature wheat embryos (cv Cadenza) as described below.

#### 2.2.2. *Ta-eIF4E/Ta-eIF(iso)4E*

To express five sgRNAs per target gene, we used sgRNA-tRNA-arrays which were constructed using a modified cloning strategy based on the report by Xie et al. [18]. In all cases, we used the Gly-tRNA sequence and an improved sgRNA backbone [19].

To target *Ta-eIF4E*, six PCRs were performed using the Q5 proof-reading DNA polymerase (NEB) with the vector pUC57-R504 (kindly provided by Alison Huttly, Rothamsted Research, Harpenden, UK) as template and primer pairs FH187/FH188, FH189/190, FH191/192, FH193/194, FH195/196 and FH197/198 (Table S1). Gel-extracted PCR products were assembled in a cut-ligation reaction using BsaI-HFv2 (New England Biolabs, Hitchin, UK) into vector pRRES208.482 (kindly provided by Alison Huttly, Rothamsted Research, Harpenden, UK) for expression under the OsU3 promoter using previously described reaction conditions [20], resulting in the pFH11 construct (Figure S4).

Similarly, to target *Ta-eIF(iso)4E*, six PCRs were performed using the vector pUC57-R504 with primer pairs FH187/FH199, FH200/FH201, FH202/FH203, FH204/FH205, FH206/FH207 and FH208/FH198 (Table S1) and the PCR amplicons were cut-ligated into pRRES208.482 using BsaI-HFv2, resulting in the construct pFH12 (Figure S5).

pFH11 was combined with pFH23 [20], encoding wheat codon-optimised Cas9 placed under the maize ubiquitin promoter (ZmUbiPr::SpCas9), and pRRes1.111 [17] encoding the *bar* selectable marker. All three plasmids were co-delivered into immature wheat embryos (cvs Cezanne, Goncourt and Prevert) as described below.

pFH12 was combined with pFH23 [20], encoding wheat codon-optimised Cas9 placed under the maize ubiquitin promoter (ZmUbiPr::SpCas9), and pRRes1.111 [17] encoding the *bar* selectable marker. All three plasmids were co-delivered into immature wheat embryos (cvs Cezanne, Goncourt and Prevert) as described below.

### 2.3. Growth of Donor Plants

The following bread wheat varieties were used for transformation: Cadenza (spring), Cezanne (winter), Goncourt (winter) and Prevert (winter).

Plants of each variety were grown in controlled environment rooms at 18 °C/15 °C day/night temperatures and ~700 µM PAR for a 16 h photoperiod. The winter varieties were initially given an 8-week vernalisation phase at 4–5 °C with ~150 µM PAR for an 8 h photoperiod.

### 2.4. Transformation

Wheat embryos of all varieties were transformed via particle bombardment essentially as previously described [21].

Donor plants were grown as above for 10–12 weeks to provide immature embryos which were isolated at 12–16 days post anthesis (dpa). The shoot/root axis was removed and the immature scutella were plated ~30 per plate on the induction medium [21], and used as target tissue, giving one day pre-culture at 22 °C, dark, prior to bombardment.

Then, 0.6 µm gold particles (BioRad Laboratories Ltd., Watford, UK) were coated with plasmid DNA as specified above and co-bombarded into tissues of the relevant wheat varieties using a rupture pressure of 650 psi and 28.5″ Hg vacuum. Following bombardment, the embryos were cultured and selected using glufosinate ammonium and putative transgenic plantlets were transferred to glasshouse conditions (all according to Sparks and Doherty [21]).

In the case of *TaBAK1-2*, tissue culture regenerated plants were screened for the presence of transforming plasmids using the following PCR primers (see Table S1): UbiPro4 + WheatCas9R1 to test for pCas9-GFP, M13F + M13R—for sgRNA plasmids (one or more) and Bar1 + Bar2—for pRRes1.111.

In the case of *Ta-eIF4E* and *Ta-eIF(iso)4E*, tissue-culture-regenerated plants were screened for the presence of transforming plasmids using the following PCR primers (see Table S1): UbiPro4 + FH147 to test for pFH23, FH209 + FH168 to test for pFH11, FH209 + FH210 to test for pFH12 and Bar1 + Bar2 to test for pRRes1.111.

All plants regenerated after selection on glufosinate were screened for CRISPR/Cas-induced indels using the PCR band shift assay [22], whether PCR-positive or -negative for plasmids used for transformation.

### 2.5. Analysis of CRISPR/Cas-Induced Mutations

#### 2.5.1. *TaBAK1-2*

**Figure 1:**
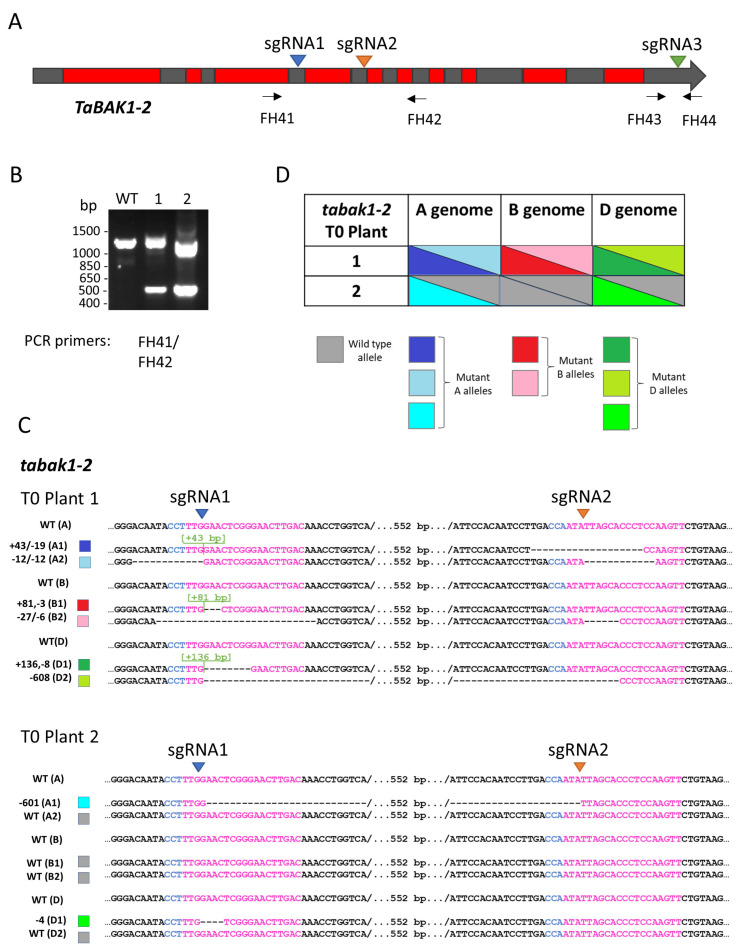
Targeted mutagenesis of *TaBAK1-2*. (**A**) Cartoon showing locations of sgRNA target sites, (**B**) PCR genotyping of CRISPR/Cas-mutagenised T0 lines, (**C**) mutant *tabak1-2* alleles identified by Sanger sequencing in T0 plants and (**D**) allele composition of *tabak1-2* T0 plants. PCR primers used for genotyping are shown as arrows.

Primary (T0) transformants were analysed for mutations in the *TaBAK1-2* gene using the PCR band shift assay with the following primers (Table S1 and Figure 1A): FH41/FH44 (amplifying across all three sgRNA targets), FH41/FH42 (amplifying across sgRNA1 and 2 targets) and primers FH43/FH44 (amplifying across the sgRNA3 target). If amplicon band shifts were visible, target genes were amplified again using the Q5 DNA polymerase (New England Biolabs) with the same primer pairs as before. The PCR products were sub-cloned using the Zero Blunt™ TOPO™ PCR Cloning Kit (Thermo Fisher Scientific, Waltham, MA, USA) and multiple single clones were Sanger-sequenced (Eurofins Genomics, Wolverhampton, UK) to detect and analyse mutations in all subgenomes.

In the case of *TaBAK1-2*, allele profiling was performed by PCR for T1 progeny lines derived from one of the T0 transformants. This was possible because each of the *TaBAK1-2* alleles from all three subgenomes carried distinct indels that resulted in clearly distinguishable migration patterns of PCR products.

#### 2.5.2. *Ta-eIF4E* and *Ta-eIF(iso)4E*

Primary (T0) transformants were initially screened for CRISPR/Cas-induced mutations in the *Ta-eIF4E* and *Ta-eIF(iso)4E* genes using the PCR band shift assay. For this, the target genes were amplified using DreamTaq DNA polymerase (ThermoFisher Scientific) and the following primers (Table S1): FH221 + FH46 (full gene), FH221 + FH445 (5′ part of the gene) and FH447 + FH46 (3′ part of the gene) in plants transformed with the vector pFH11 (in the case of targeting *Ta-eIF4E*; Figure S16) or primers FH431 + FH224 (full gene), FH431 + FH59 (5′ part of gene) and FH56 + FH57 (3′ part of gene) in plants transformed with the vector pFH12 (in the case of targeting *Ta-eIF(iso)4E*; Figure S17). If band shifts were detected, the respective gene fragments were amplified using Q5 DNA polymerase and subcloned. Multiple single clones were Sanger-sequenced, as described above for *TaBAK1-2*.

## 3. Results

### 3.1. Targeted Mutagenesis of the TaBAK1-2 Gene

We used the CRISPR/Cas system to target three *TaBAK1-2* homoeologues located on chromosome 2: *TraesCS2A02G343100* (*TaBAK1-2A*), *TraesCS2B01G340700* (*TaBAK1-2B*) and *TraesCS2D02G321400* (*TaBAK1-2D*). All three homoeologues were targeted at three conserved sgRNA target sites within exons 4 (sgRNA1), 5 (sgRNA2) and 11 (sgRNA3) (Figure 1A). We transformed wheat immature embryos (cv Cadenza) with DNA constructs expressing CRISPR/Cas reagents, as described in Materials and Methods, and regenerated 30 T0 primary transgenic lines. We then genotyped the T0 lines for the presence of CRISPR/Cas-induced indels using the PCR band shift assay [22]. Out of the 30 T0 lines, two showed a clear PCR band shift indicating the presence of deletions of around 600 bp (Figure 1B). To identify *TaBAK1-2* alleles present in both T0 plants, we subcloned the PCR amplicons into a high copy number vector and sequenced individual clones by Sanger. The T0 plant 1 turned out to be a triple-biallelic carrying indels in all six copies of *TaBAK1-2*, while the plant 2 carried heterozygous mutations in *TaBAK1-2A* and *TaBAK1-2D* and no mutations in *TaBAK1-2B* (Figure 1C,D). The three insertions identified in the T0 plant 1 (43 bp, 81 bp and 136 bp; Figure 1C) proved to be fragments of the pCas9-GFP plasmid. Such insertion events at CRISPR/Cas target sites were previously reported in potato and rice [23,24,25]. It should be noted that PCR amplicons shown in Figure 1B were produced using primers amplifying across the sgRNA1 and sgRNA2 target sites. We also separately amplified across the sgRNA3 target in both T0 plants but did not detect any mutations at this site.

As part of the mutant analysis, we translated the coding sequences from all *tabak1-2* mutant alleles and aligned them to the corresponding wild type sequences (Figures S8–S15). As a result, we were able to conclude that the T0 plant 1 alleles A1, D1 and D2, and T0 plant 2 allele D1 carried frame-shift mutations that were likely to result in complete loss of the gene function, while indels in the rest of the mutant alleles did not put the coding sequence out of frame and thus, potentially, did not lead to gene knockout. 

We selected the *tabak1-2* T0 line 1 for further analysis as this line showed mutations in all six copies of the *TaBAK1-2* gene.

**Figure 2:**
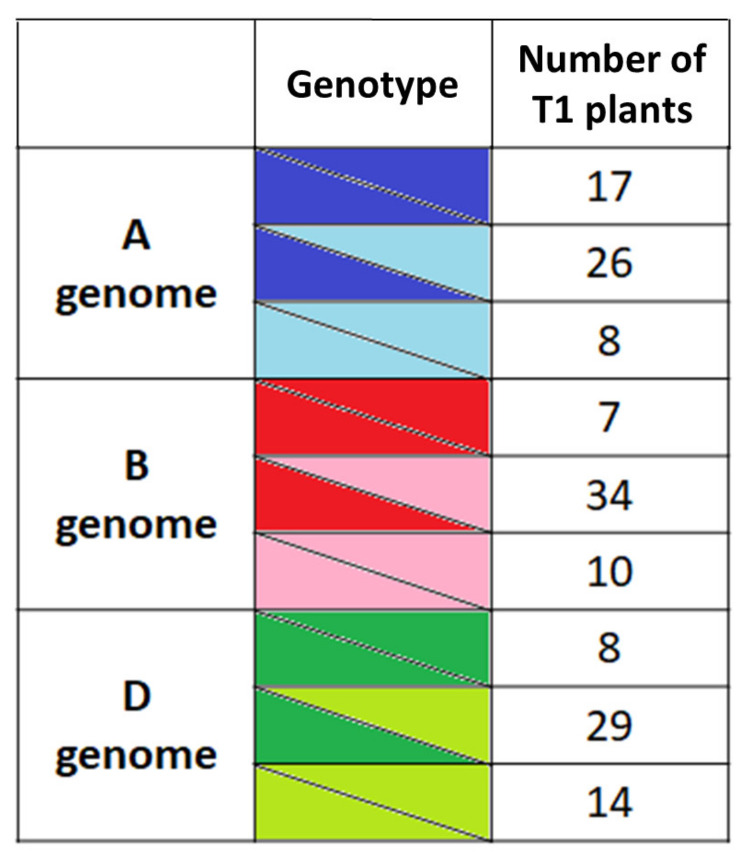
CRISPR/Cas-induced *tabak1-2* mutant alleles were inherited by the next generation. The table shows allele distribution among T1 progeny of the T0 plant 1.

To check if the mutations present in the *tabak1-2* T0 line 1 could be transmitted through the germline and inherited by the next generation, we PCR-genotyped 52 T1 progeny plants derived from this line. The genotyping data clearly indicated inheritance of all six mutant alleles (Figure 2, Figures S6 and S7). Out of 52 T1 lines, five were triple-homozygous (plants 4, 19, 23, 43 and 52; Figure S7).

### 3.2. Targeted Mutagenesis of the Ta-eIF4E and Ta-eIF(iso)4E Genes

**Figure 3:**
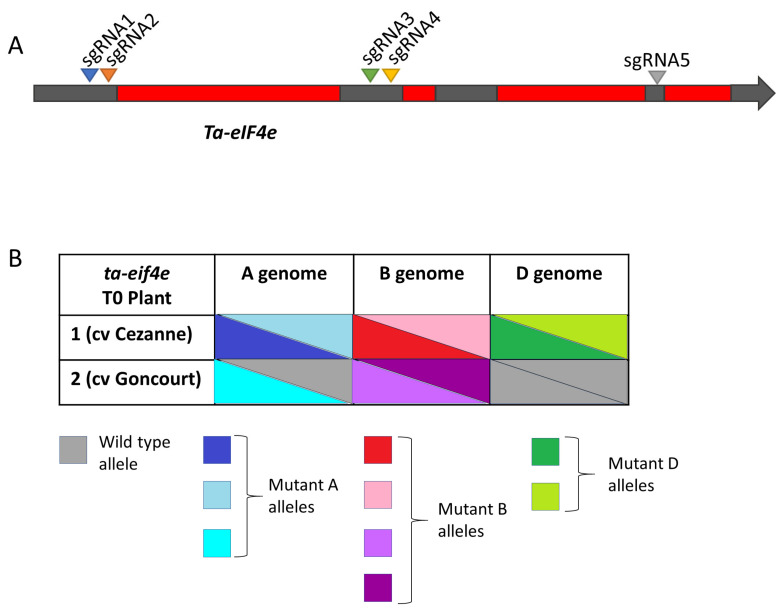
Targeted mutagenesis of *Ta-eIF4e*. (**A**) Cartoon showing locations of sgRNA target sites and (**B**) allele composition of *ta-eif4e* T0 plants.

**Figure 4:**
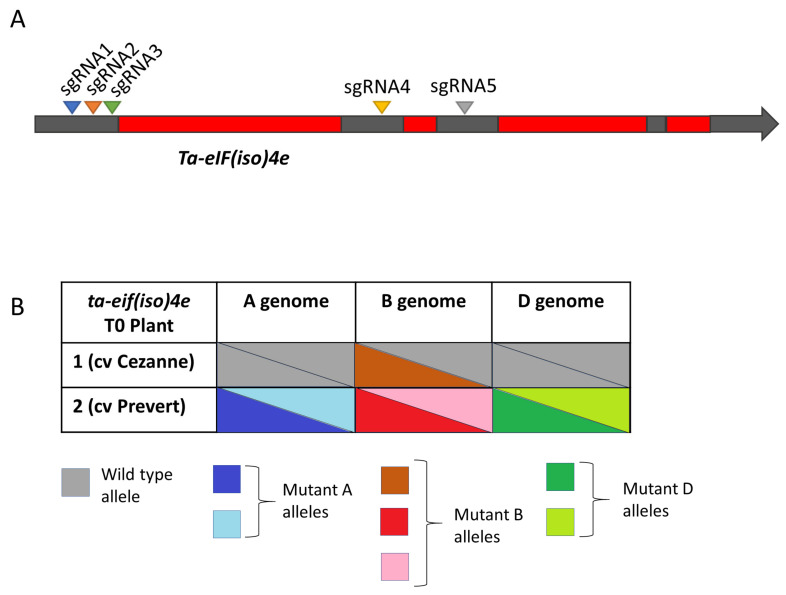
Targeted mutagenesis of *Ta-eIF(iso)4e*. (**A**) Cartoon showing locations of sgRNA target sites and (**B**) allele composition of *ta-eif(iso)4e* T0 plants.

We targeted both *Ta-eIF4E* (homoeologues *TraesCS3A02G521500*, *TraesCS3B02G591300* and *TraesCS3D02G527800*) and *Ta-eIF(iso)4E* (homoeologues *TraesCS1A02G149200*, *TraesCS1B02G167100* and *TraesCS1D02G146500*) genes with five sgRNAs each (Figure 3A and Figure 4A, respectively), in three winter cultivars of common wheat (Cezanne, Goncourt and Prevert), which are susceptible to WSSMV (Dragan Perovic, personal communication).

In total, we screened 49 T0 plants for *Ta-eIF4E* (40, 8 and 1 from cvs Cezanne, Goncourt and Prevert transformations, respectively), and 40 T0 plants for *Ta-eIF(iso)4E* (30, 5 and 5 from cvs Cezanne, Goncourt and Prevert transformations, respectively). Genotyping identified two T0 plants carrying CRISPR/Cas-induced indels in *Ta-eIF4E* (cvs Cezanne and Goncourt; Figure 3B, Figures S16 and S18) and two T0 plants with indels in *Ta-eIF(iso)4E* (cvs Cezanne and Prevert; Figure 4B, Figures S17, S24 and S25). Two out of the four T0 plants were triple-biallelic: *ta-eif4e* T0 plant 1 (cv Cezanne; Figure 3B and Figure S18) and *ta-eif(iso)4e* T0 plant 2 (cv Prevert; Figure 4B and Figure S25).

As in the case of *TaBAK1-2*, we detected CRISPR/Cas-induced insertions in *ta-eif4e* and *ta-eif(iso)4e* T0 lines ranging from 242 bp to 592 bp (Figures S18, S24 and S25). The inserted DNA was derived from plasmids used for transformation, wheat genomic DNA, bacterial DNA or combinations of those.

We also translated the coding sequences from all *ta-eif4e* and *ta-eif(iso)4e* mutant alleles and aligned them to the corresponding wild type protein sequences (Figures S19–S23 and Figures S26–S29, respectively). The alignments indicated that all *ta-eif4e* and *ta-eif(iso)4e* alleles, except the B2 allele in *ta-eif4e* T0 plant 2 (Figures S18 and S23B), carried frame-shift mutations that were likely to cause loss of the gene function.

## 4. Discussion

During this study we generated *tabak1-2* lines carrying different combinations of mutant *tabak1-2a, tabak1-2b* and *tabak1-2d* alleles, including multiple homozygous lines (Figure S7), in the spring wheat cultivar Cadenza, which is relatively easy to transform and whose genome has now been sequenced as part of the 10+ Wheat Genomes Project (http://www.10wheatgenomes.com/, accessed on 16 July 2021). BAK1 acts as a coreceptor for a number of pattern recognition receptors (PRRs) involved in pattern-triggered immunity (PTI) responses in plants [5]. As mentioned in the Results section, some of the generated *tabak1-2* alleles carry frame-shift mutations and are likely to behave as null, while the rest carry in-frame indels that might not fully disrupt the gene function. Nevertheless, the latter type of allele carries rather large deletions/insertions within the coding regions that hopefully compromise the gene function to significant extent but further studies would be needed to verify this. We therefore hope the generated *tabak1-2* lines will become a useful genetic resource for the research community working on molecular mechanisms of plant-microbe interactions in wheat.

To generate the *ta-eif4e* and *ta-eif(iso)4e* lines, we needed to perform CRISPR/Cas mutagenesis in wheat cultivars, which are known to be susceptible to WSSMV. We chose winter wheat varieties Cezanne, Goncourt and Prevert based on the available phenotypic data (Dragan Perovic, personal communication). Generally, winter cultivars are more difficult to transform as they require vernalisation and the transformation rates are lower compared to spring varieties and, as part of the study, we optimised the transformation procedure for them (see Materials and Methods). Since there are only few published examples of genome editing in winter wheat [26,27], our work will be of interest to researchers working on winter wheat transformation and genome editing.

As already mentioned in the Results section, all but one mutant allele in *ta-eif4e* and *ta-eif(iso)4e* lines carry frame-shift mutations and, therefore, are likely to be loss-of-function and, consequently, a source of resistance to WSSMV.

To be able to evaluate the mutants for enhanced resistance to WSSMV, phenotypic characterisation of the lines would need to be carried out in a follow-up study. Since WSSMV is transmitted by *Polymyxa graminis* [7], a soil-borne filamentous microorganism infecting wheat roots, it is very difficult to perform WSSMV pathotests under glasshouse conditions. On the other hand, GM field trials in the south of Europe, where WSSMV can be found, are problematic right now due to the policy restrictions and significant public opposition. We are hopeful the situation will change at some point in the future. It should be noted, that since some of the *ta-eif4e* and *ta-eif(iso)4e* mutant alleles contain inserted fragments of transgenic or wheat genomic DNA, plants carrying them cannot be treated as transgene-free genome edited but rather GM lines.

The insertion of relatively large fragments of plasmid DNA at some of the sgRNA target sites was a common issue with most of our CRISPR/Cas-mutagenised lines. Similar observations were previously made in potato and rice [23,24,25], and in the case of rice particle bombardment was used as the method for transformation. Particle bombardment involves the release of a high number of plasmid copies into the immature embryo and they can integrate into double strand breaks in the chromosomal DNA. For gene function studies, inserting a large fragment of DNA is a good way to disrupt the gene function and the high copy number of plasmid DNA encoding CRISPR/Cas components might even enhance the editing efficiency. However, if commercialisation of a CRISPR-edited line is desired one might consider *Agrobacterium*-mediated transgene delivery methods and use of a low copy number vector [28] to avoid integration of transgenic DNA at CRISPR/Cas target sites and, consequently, GM regulatory issues. It has to be noted though that not all plants are amenable to *Agrobacterium*-mediated transformation and that particle bombardment has other advantages compared to this method, such as the possibility to deliver proteins or RNA.

Due to its hexaploid genome, screening for CRISPR/Cas-induced mutations in wheat is challenging as e.g., analysis of Sanger-sequenced PCR amplicons generated from all six alleles is in most cases difficult due to small sequence variation between the homoeologues. We therefore chose the well-established PCR band shift assay [22] to detect large deletions between two target sites. This allowed us to recover triple-biallelic mutants for all three targeted genes. It should however be noted that the overall mutation frequencies in our experiments are probably even higher, as our mutation screening approach would have missed small indels present at single target sites. We expect that next generation sequencing (NGS) approaches will become the gold standard for mutation detection in polyploid organisms in the near future, as these are able to detect small indels amongst all homoeologues and target sites.

Optimisation of the CRISPR/Cas components is also important for boosting gene editing efficiencies, especially in genetically complex plants, such as wheat. In a previous study, we could demonstrate that the efficiency of different Cas9 versions varied in wheat protoplasts (cv Cadenza), probably due to differences in codon optimisation or nuclear localisation signals [20]. As part of that study, we also found one Cas9 variant, which showed a drastically higher efficiency compared to other tested Cas9 versions [20]. In future experiments, researchers might want to use that Cas9 variant to boost editing efficiencies in wheat even further.

## Figures and Tables

**Table 1:** CRISPR/Cas targets.

*TaBAK1-2*	sgRNA1	GTCAAGTTCCCGAGTTCCAA
	sgRNA2	AACTTGGAGGGTGCTAATAT
	sgRNA3	GATCCAGTCGTTGTTTCGCG
*Ta-eIF4E*	sgRNA1	GCTCCCACATTCAACTTGCT
	sgRNA2	GTTGTCGAACCAGAAGGTCC
	sgRNA3	GAAGGTGTGGATGGGGTGGA
	sgRNA4	GATGGTCCATTTACCGCCAT
	sgRNA5	GAAGGAGTTTCTGGACTACA
*Ta-eIF(iso)4E*	sgRNA1	GAACTCTTCGACGGTGTCGA
	sgRNA2	GGCTGGGGTAGAACCAAAGT
	sgRNA3	GACAGGATAAGCTTTCATTA
	sgRNA4	GGTCTGGATGTCGTACCAGA
	sgRNA5	GGTCGAAGCTGCGCTCCCGG

## Supplementary Materials

The following are available online at https://www.mdpi.com/article/10.3390/plants10071481/s1, Table S1: Primers used for genotyping of transgenic lines, Figure S1: pUC19_rice_sgRNA_v2_sgRNA1 construct map and insert sequence, Figure S2: pUC19_rice_sgRNA_v2_sgRNA2 construct map and insert sequence, Figure S3: pUC19_rice_sgRNA_v2_sgRNA3 construct map and insert sequence, Figure S4: pFH11 construct map and insert sequence, Figure S5: pFH12 construct map and insert sequence, Figure S6: PCR-genotyping of T1 progeny of tabak1-2 T0 plant 1 with FH227 + FH228 primers (A) and FH227 + FH230 primers (B), Figure S7: *TaBAK1-2* allele distribution among T1 progeny of *tabak1-2* T0 plant 1, Figure S8: Protein alignment of the *tabak1-2* T0 plant 1 A1 allele to the WT, Figure S9: Protein alignment of the *tabak1-2* T0 plant 1 A2 allele to the WT, Figure S10: Protein alignment of the *tabak1-2* T0 plant 1 B1 allele to the WT, Figure S11: Protein alignment of the *tabak1-2* T0 plant 1 B2 allele to the WT, Figure S12: Protein alignment of the *tabak1-2* T0 plant 1 D1 allele to the WT, Figure S13: Protein alignment of the *tabak1-2* T0 plant 1 D2 allele to the WT, Figure S14: Protein alignment of the *tabak1-2* T0 plant 2 A1 allele to the WT, Figure S15: Protein alignment of the *tabak1-2* T0 plant 2 D1 allele to the WT, Figure S16: PCR genotyping of the *ta-eif4e* T0 plants 1 and 2 (cvs Cezanne and Goncourt, respectively), Figure S17: PCR genotyping of the *ta-eif(iso)4e* T0 plants 1 and 2 (cvs Cezanne and Prevert, respectively), Figure S18: Alignments showing CRISPR/Cas-induced indels in *Ta-eIF4e* homoeologues in two *ta-eif4e* T0 plants (cvs Cezanne and Goncourt), Figure S19: Protein alignments of the *ta-eif4e* T0 plant 1 A1 (A) and A2 (B) alleles to the WT, Figure S20: Protein alignments of the *ta-eif4e* T0 plant 1 B1 (A) and B2 (B) alleles to the WT, Figure S21: Protein alignments of the *ta-eif4e* T0 plant 1 D1 (A) and D2 (B) alleles to the WT, Figure S22: Protein alignment of the *ta-eif4e* T0 plant 2 A2 allele to the WT, Figure S23: Protein alignments of the *ta-eif4e* T0 plant 2 B1 (A) and B2 (B) alleles to the WT, Figure S24: Alignment showing CRISPR/Cas-induced indels in *Ta-eIF(iso)4e* homoeologues in the *ta-eif(iso)4e* T0 plant 1 (cv Cezanne), Figure S25: Alignment showing CRISPR/Cas-induced indels in *Ta-eIF(iso)4e* homoeologues in the *ta-eif(iso)4e* T0 plant 2 (cv Prevert), Figure S26: Protein alignment of the *ta-eif(iso)4e* T0 plant 1 B1 allele to the WT, Figure S27: Protein alignments of the *ta-eif(iso)4e* T0 plant 2 A1 (A) and A2 (B) alleles to the WT, Figure S28: Protein alignments of the *ta-eif(iso)4e* T0 plant 2 B1 (A) and B2 (B) alleles to the WT, Figure S29: Protein alignments of the *ta-eif(iso)4e* T0 plant 2 D1 (A) and D2 (B) alleles to the WT.
