# Supplementary material for: Efficient CRISPR/Cas-Mediated Targeted Mutagenesis in Spring and Winter Wheat Varieties
Source: Plants (Basel). 2021 Jul 19;10(7):1481. doi: 10.3390/plants10071481 (PMC8309376; doi:10.3390/plants10071481)
Supplement: Supplementary file 1 [file plants-10-01481-s001.zip › plants-1283803-supplementary.pdf]

Table S1
Primers used for genotyping of transgenic lines.

Primer	5'-3' Sequence
UbiPro4	TTTAGCCCTGCCTTCATACG
WheatCas9R1	GGATTAAGGTCGCCTTCGATGAGG
M13F	GTAAAACGACGGCCAGT
M13R	CAGGAAACAGCTATGAC
Bar1	GTCTGCACCATCGTCAACC
Bar2	GAAGTCCAGCTGCCAGAAAC
FH41	GAGATTTCTGCTGAGATCCTACAT
FH42	GGATTATTACCAAAACTGTGACAAT
FH43	CTGAAAGAGAAGAAGGTGGAGAT
FH44	GCTATCTGTCGGTCATCATGTAC
FH46	TCAAACGGTGTAGCGGTTCTT
FH56	GTATGATCAGATTTTCCGTCCG
FH57	TGAACAGCTTCGTTACTGGCAG
FH59	TGTCCAAAAGCAATCCGACC
FH147	GATGCGGAAGGTCAGGATCT
FH168	AGCAAGTTGAATGTGGGAGC
FH187	GTGGTCTCCGGCAACAAAGCACCAGTGGTCT
FH188	TAGGTCTCAATGTGGGAGCTGCACCAGCCGGGAATC
FH189	GTGGTCTCCACATTCAACTTGCTGTTTCAGAGCTATGCTGGGAACA
FH190	TAGGTCTCAGTTCGACAACTGCACCAGCCGGGAATC
FH191	GTGGTCTCCGAACCAGAAGGTCCGTTTCAGAGCTATGCTGGGAACA
FH192	TAGGTCTCACCACACCTTCTGCACCAGCCGGGAATC
FH193	GTGGTCTCCGTGGATGGGGTGGAGTTTCAGAGCTATGCTGGGAACA
FH194	TAGGTCTCAATGGACCATCTGCACCAGCCGGGAATC
FH195	GTGGTCTCCCCATTTACCGCCATGTTTCAGAGCTATGCTGGGAACA
FH196	TAGGTCTCAAACTCCTTCTGCACCAGCCGGGAATC
FH197	GTGGTCTCCAGTTTCTGGACTACAGTTTCAGAGCTATGCTGGGAACA
FH198	GTGCGGTCTCCAAACAAAAAAAAAGCACCGACTCGGTGCCACT
FH199	TAGGTCTCACGAAGAGTTCTGCACCAGCCGGGAATC
FH200	GTGGTCTCCTTCGACGGTGTCGAGTTTCAGAGCTATGCTGGGAACA
FH201	TAGGTCTCATACCCCAGCCTGCACCAGCCGGGAATC
FH202	GTGGTCTCCGGTAGAACCAAAGTGTTTCAGAGCTATGCTGGGAACA
FH203	TAGGTCTCATTATCCTGTCTGCACCAGCCGGGAATC
FH204	GTGGTCTCCATAAGCTTTCATTAGTTTCAGAGCTATGCTGGGAACA
FH205	TAGGTCTCACATCCAGACCTGCACCAGCCGGGAATC
FH206	GTGGTCTCCGATGTCGTACCAGAGTTTCAGAGCTATGCTGGGAACA
FH207	TAGGTCTCAGCTTCGACCTGCACCAGCCGGGAATC
FH208	GTGGTCTCCAAGCTGCGCTCCCGGGTTTCAGAGCTATGCTGGGAACA
FH209	TGAAGCCTTTCAGGACATGTAT
FH210	CACTATAGGGCGAATTGGAGA
FH221	TAGAAAAGCCCGCGACTTTC
FH224	ATTAGCTGAGGCGGTTCGAATCT
FH227	TGTAAAACGACGGCCAGTTTTGGCGCTCTCATTATGTAT
FH228	CCTCTCTATGGGCAGTCGGTGATAGGAATCGCAACTTTAAGAGTT
FH230	CCTCTCTATGGGCAGTCGGTGATCATGCCCACAGCATATCCT
FH431	TCCCCATCCCAAAACCCTCG
FH445	TGTTCACCAATCATTGCCAGC
FH447	ATTTACCAGATAAGCATTGGCAA

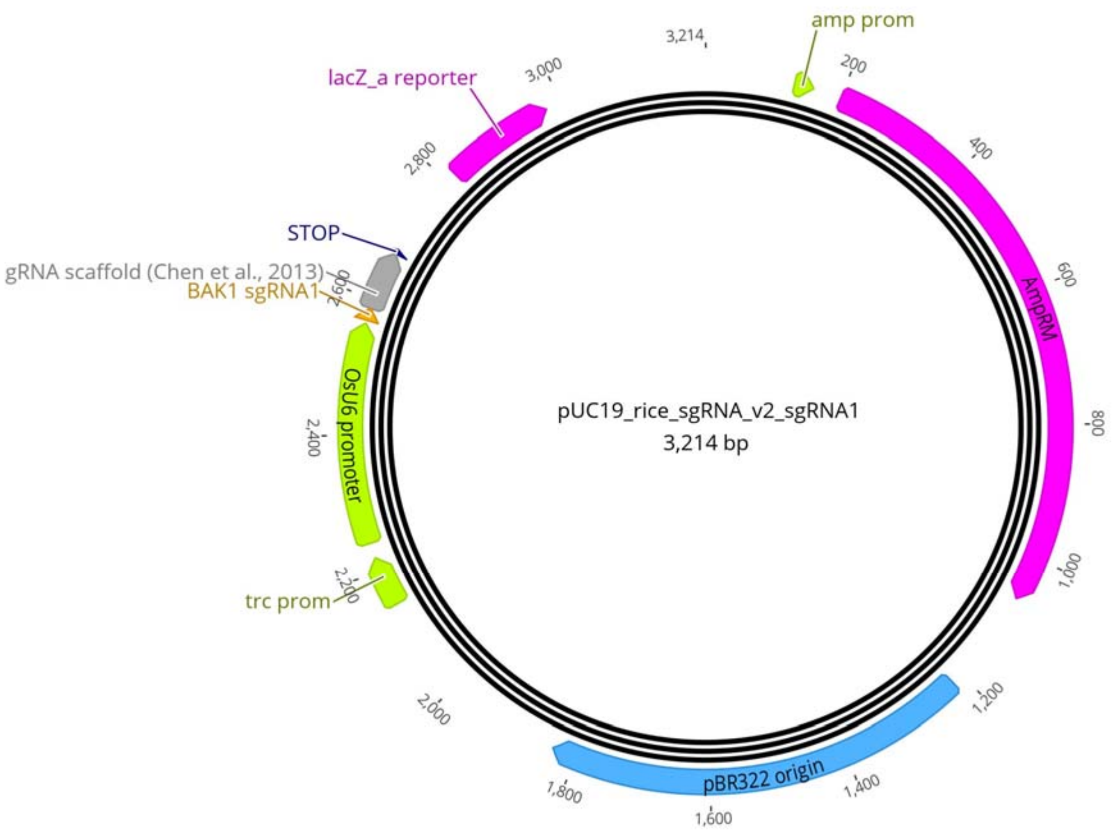

AAGAACGAACTAAGCCGGACAAAAAAAGGAGCACATATACAAACCGGTTTTATTCATGAATGGTCACGATGGATGATGG
GGCTCAGACTTGAGCTACGAGGCCGCAGGCGAGAGAAGCCTAGTGTGCTCTCTGCTTGTTTGGGCCGTAACGGAGGATA
CGGCCGACGAGCGTGTACTACCGCGCGGGATGCCGCTGGGCGCTGCGGGGGCCGTTGGATGGGGATCGGTGGGTCGCGG
GAGCGTTGAGGGGAGACAGGTTTAGTACCACCTCGCCTACCGAACAATGAAGAACCCACCTTATAACCCCGCGCGCTGC
CGCTTGTGTTGTCAAGTTCCCGAGTTCCAAGTTTAAGAGCTATGCTGGAAACAGCATAGCAAGTTTAAATAAGGCTAGT
CCGTTATCAACTTGAAAAAGTGGCACCGAGTCGGTGCTTTTTTTT

OsU6 promoter
gRNA scaffold (Chen et al., 2013)
PolyT STOP
Target sequence

Figure S1
pUC19_rice_sgRNA_v2_sgRNA1 construct map and insert sequence.

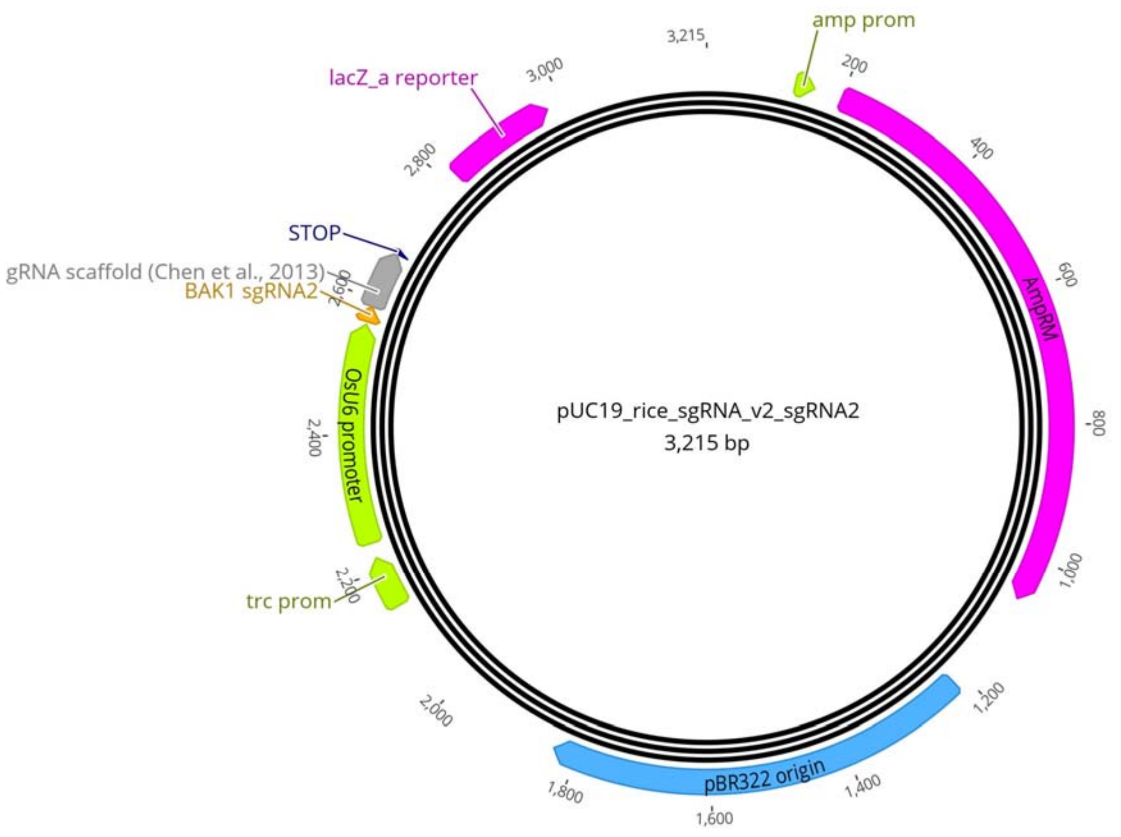

AAGAACGAACTAAGCCGGACAAAAAAAGGAGCACATATACAAACCGGTTTTATTCATGAATGGTCACGATGGATGATGG
GGCTCAGACTTGAGCTACGAGGCCGCAGGCGAGAGAAGCCTAGTGTGCTCTCTGCTTGTTTGGGCCGTAACGGAGGATA
CGGCCGACGAGCGTGTACTACCGCGCGGGATGCCGCTGGGCGCTGCGGGGGCCGTTGGATGGGGATCGGTGGGTCGCGG
GAGCGTTGAGGGGAGACAGGTTTAGTACCACCTCGCCTACCGAACAATGAAGAACCCACCTTATAACCCCGCGCGCTGC
CGCTTGTGTTGAACTTGGAGGGTGCTAATATGTTTAAGAGCTATGCTGGAAACAGCATAGCAAGTTTAAATAAGGCTAG
TCCGTTATCAACTTGAAAAAGTGGCACCGAGTCGGTGCTTTTTTTT

OsU6 promoter
gRNA scaffold (Chen et al., 2013)
PolyT STOP
Target sequence

Figure S2
pUC19_rice_sgRNA_v2_sgRNA2 construct map and insert sequence.

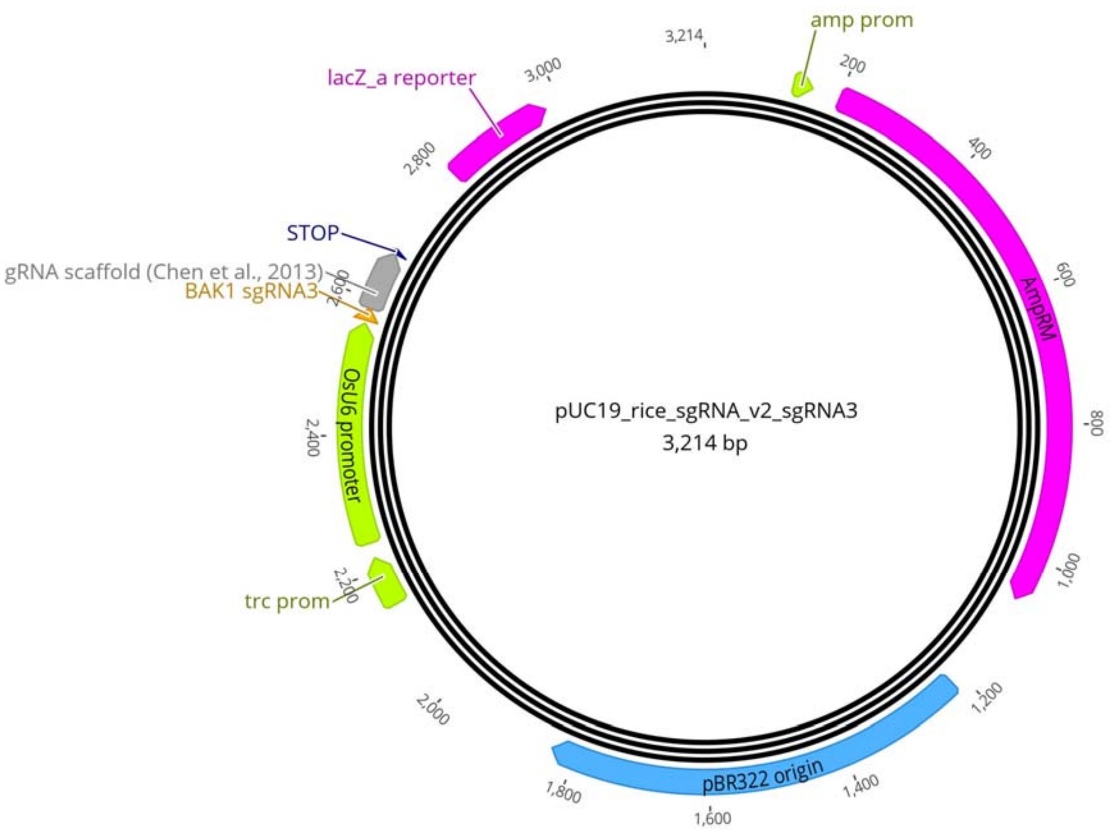

AAGAACGAACTAAGCCGGACAAAAAAAGGAGCACATATACAAACCGGTTTTATTCATGAATGGTCACGATGGATGATGG
GGCTCAGACTTGAGCTACGAGGCCGCAGGCGAGAGAAGCCTAGTGTGCTCTCTGCTTGTTTGGGCCGTAACGGAGGATA
CGGCCGACGAGCGTGTACTACCGCGCGGGATGCCGCTGGGCGCTGCGGGGGCCGTTGGATGGGGATCGGTGGGTCGCGG
GAGCGTTGAGGGGAGACAGGTTTAGTACCACCTCGCCTACCGAACAATGAAGAACCCACCTTATAACCCCGCGCGCTGC
CGCTTGTGTTGATCCAGTCGTTGTTTCGCGGTTTAAGAGCTATGCTGGAAACAGCATAGCAAGTTTAAATAAGGCTAGT
CCGTTATCAACTTGAAAAAGTGGCACCGAGTCGGTGCTTTTTTTT

OsU6 promoter
gRNA scaffold (Chen et al., 2013)
PolyT STOP
Target sequence

Figure S3
pUC19_rice_sgRNA_v2_sgRNA3 construct map and insert sequence.

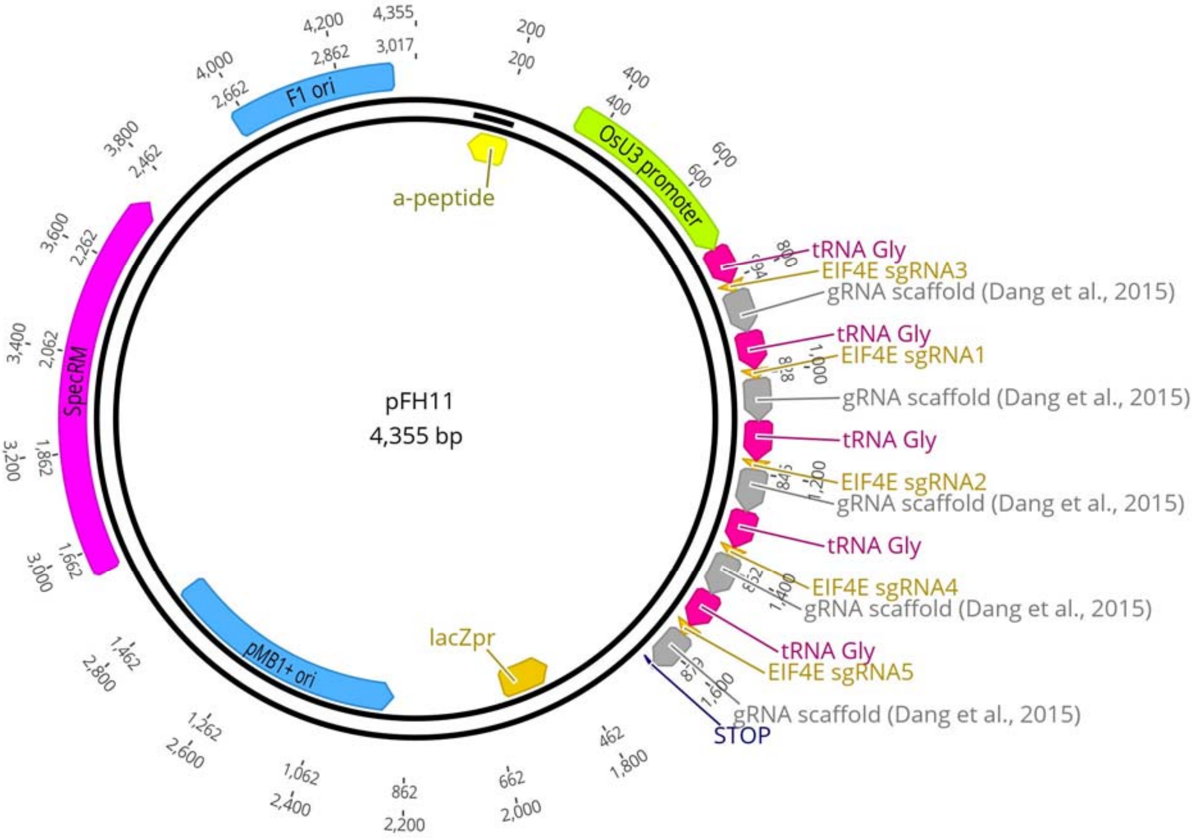

AAGCTTAAGGAATCTTTAAACATACGAACAGATCACTTAAAGTTCTTCTGAAGCAACTTAAAGTTATCAGGCATGCATG
GATCTTGGAGGAATCAGATGTGCAGTCAGGGACCATAGCACAAGACAGGCGTCTTCTACTGGTGCTACCAGCAAATGCT
GGAAGCCGGGAACACTGGGTACGTTGGAAACCACGTGATGTGAAGAAGTAAGATAAACTGTAGGAGAAAAGCATTTCGT
AGTGGGCCATGAAGCCTTTCAGGACATGTATTGCAGTATGGGCCGGCCCATTACGCAATTGGACGACAACAAAGACTAG
TATTAGTACCACCTCGGCTATCCACATAGATCAAAGCTGATTTAAAAGAGTTGTGCAGATGATCCGTGGCAACAAAGCA
CCAGTGGTCTAGTGGTAGAATAGTACCCTGCCACGGTACAGACCCGGGTTCGATTCCCGGCTGGTGCAGCTCCCACATT
CAACTTGCTGTTTCAGAGCTATGCTGGGAACAGCATAGCAAGTTGAAATAAGGCTAGTCCGTTATCAACTTGAAAAAGT
GGCACCGAGTCGGTGCAACAAAGCACCAGTGGTCTAGTGGTAGAATAGTACCCTGCCACGGTACAGACCCGGGTTCGAT
TCCCGGCTGGTGCAGTTGTCGAACCAGAAGGTCCGTTTCAGAGCTATGCTGGGAACAGCATAGCAAGTTGAAATAAGGC
TAGTCCGTTATCAACTTGAAAAAGTGGCACCGAGTCGGTGCAACAAAGCACCAGTGGTCTAGTGGTAGAATAGTACCCT
GCCACGGTACAGACCCGGGTTCGATTCCCGGCTGGTGCAGAAGGTGTGGATGGGGTGGAGTTTCAGAGCTATGCTGGGA
ACAGCATAGCAAGTTGAAATAAGGCTAGTCCGTTATCAACTTGAAAAAGTGGCACCGAGTCGGTGCAACAAAGCACCAG
TGGTCTAGTGGTAGAATAGTACCCTGCCACGGTACAGACCCGGGTTCGATTCCCGGCTGGTGCAGATGGTCCATTTACC
GCCATGTTTCAGAGCTATGCTGGGAACAGCATAGCAAGTTGAAATAAGGCTAGTCCGTTATCAACTTGAAAAAGTGGCA
CCGAGTCGGTGCAACAAAGCACCAGTGGTCTAGTGGTAGAATAGTACCCTGCCACGGTACAGACCCGGGTTCGATTCCC
GGCTGGTGCAGAAGGAGTTTCTGGACTACAGTTTCAGAGCTATGCTGGGAACAGCATAGCAAGTTGAAATAAGGCTAGT
CCGTTATCAACTTGAAAAAGTGGCACCGAGTCGGTGCTTTTTTTTT

OsU3 promoter
tRNA Gly
gRNA scaffold (Dang et al., 2015)
PolyT STOP
Target sequences

Figure S4
pFH11 construct map and insert sequence.

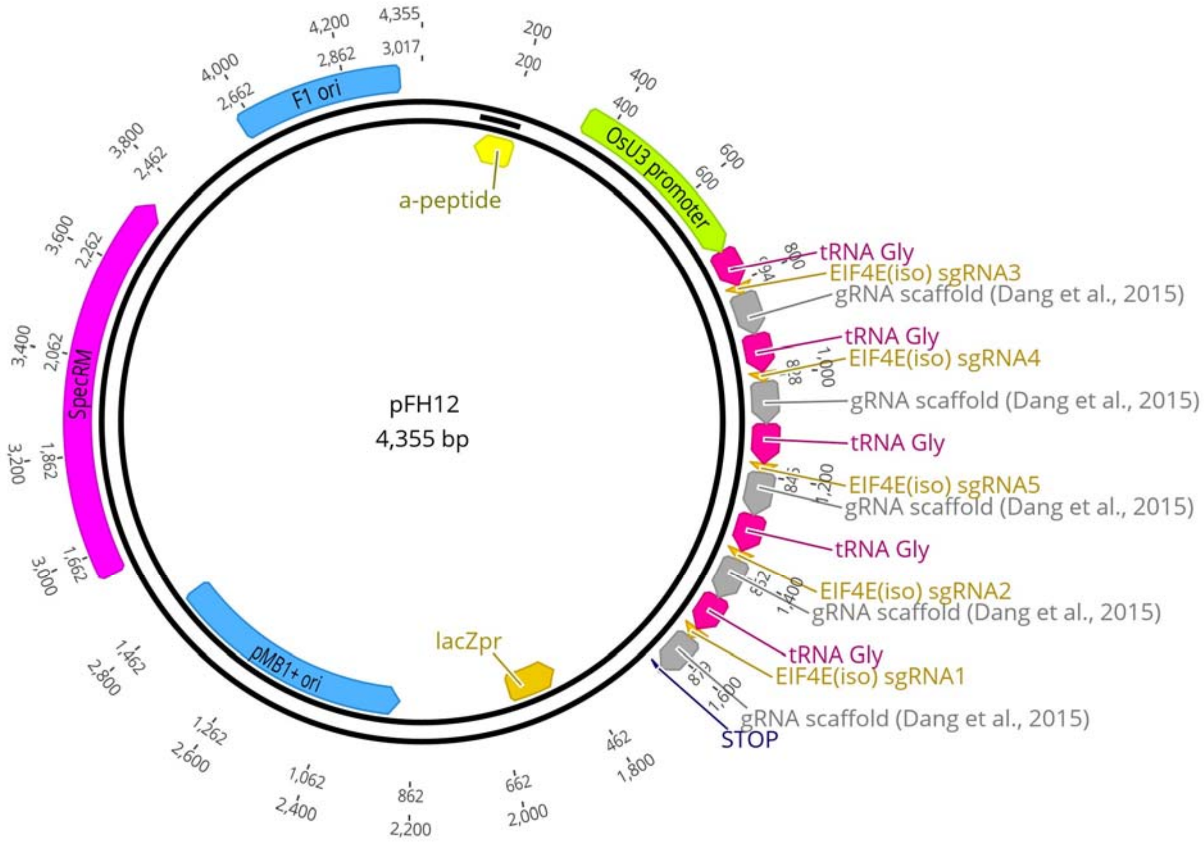


```
AAGCTTAAGGAATCTTTAAACATACGAACAGATCACTTAAAGTTCTTCTGAAGCAACTTAAAGTTATCAGGCATGCATG
GATCTTGGAGGAATCAGATGTGCAGTCAGGGACCATAGCACAAGACAGGCGTCTTCTACTGGTGCTACCAGCAAATGCT
GGAAGCCGGGAACACTGGGTACGTTGGAAACCACGTGATGTGAAGAAGTAAGATAAACTGTAGGAGAAAAGCATTTCGT
AGTGGGCCATGAAGCCTTTCAGGACATGTATTGCAGTATGGGCCGGCCCATTACGCAATTGGACGACAACAAAGACTAG
TATTAGTACCACCTCGGCTATCCACATAGATCAAAGCTGATTTAAAAGAGTTGTGCAGATGATCCGTGGCAACAAAGCA
CCAGTGGTCTAGTGGTAGAATAGTACCCTGCCACGGTACAGACCCGGGTTCGATTCCCGGCTGGTGCAGAACTCTTCGA
CGGTGTCGAGTTTCAGAGCTATGCTGGGAACAGCATAGCAAGTTGAAATAAGGCTAGTCCGTTATCAACTTGAAAAAGT
GGCACCGAGTCGGTGCAACAAAGCACCAGTGGTCTAGTGGTAGAATAGTACCCTGCCACGGTACAGACCCGGGTTCGAT
TCCCGGCTGGTGCAGGCTGGGGTAGAACCAAAGTGTTTCAGAGCTATGCTGGGAACAGCATAGCAAGTTGAAATAAGGC
TAGTCCGTTATCAACTTGAAAAAGTGGCACCGAGTCGGTGCAACAAAGCACCAGTGGTCTAGTGGTAGAATAGTACCCT
GCCACGGTACAGACCCGGGTTCGATTCCCGGCTGGTGCAGACAGGATAAGCTTTCATTAGTTTCAGAGCTATGCTGGGA
ACAGCATAGCAAGTTGAAATAAGGCTAGTCCGTTATCAACTTGAAAAAGTGGCACCGAGTCGGTGCAACAAAGCACCAG
TGGTCTAGTGGTAGAATAGTACCCTGCCACGGTACAGACCCGGGTTCGATTCCCGGCTGGTGCAGGTCTGGATGTCGTA
CCAGAGTTTCAGAGCTATGCTGGGAACAGCATAGCAAGTTGAAATAAGGCTAGTCCGTTATCAACTTGAAAAAGTGGCA
CCGAGTCGGTGCAACAAAGCACCAGTGGTCTAGTGGTAGAATAGTACCCTGCCACGGTACAGACCCGGGTTCGATTCCC
GGCTGGTGCAGGTCGAAGCTGCGCTCCCGGGTTTCAGAGCTATGCTGGGAACAGCATAGCAAGTTGAAATAAGGCTAGT
CCGTTATCAACTTGAAAAAGTGGCACCGAGTCGGTGCTTTTTTTTT
```

OsU3 promoter
tRNA Gly
gRNA scaffold (Dang et al., 2015)
PolyT STOP
Target sequences

Figure S5
pFH12 construct map and insert sequence.

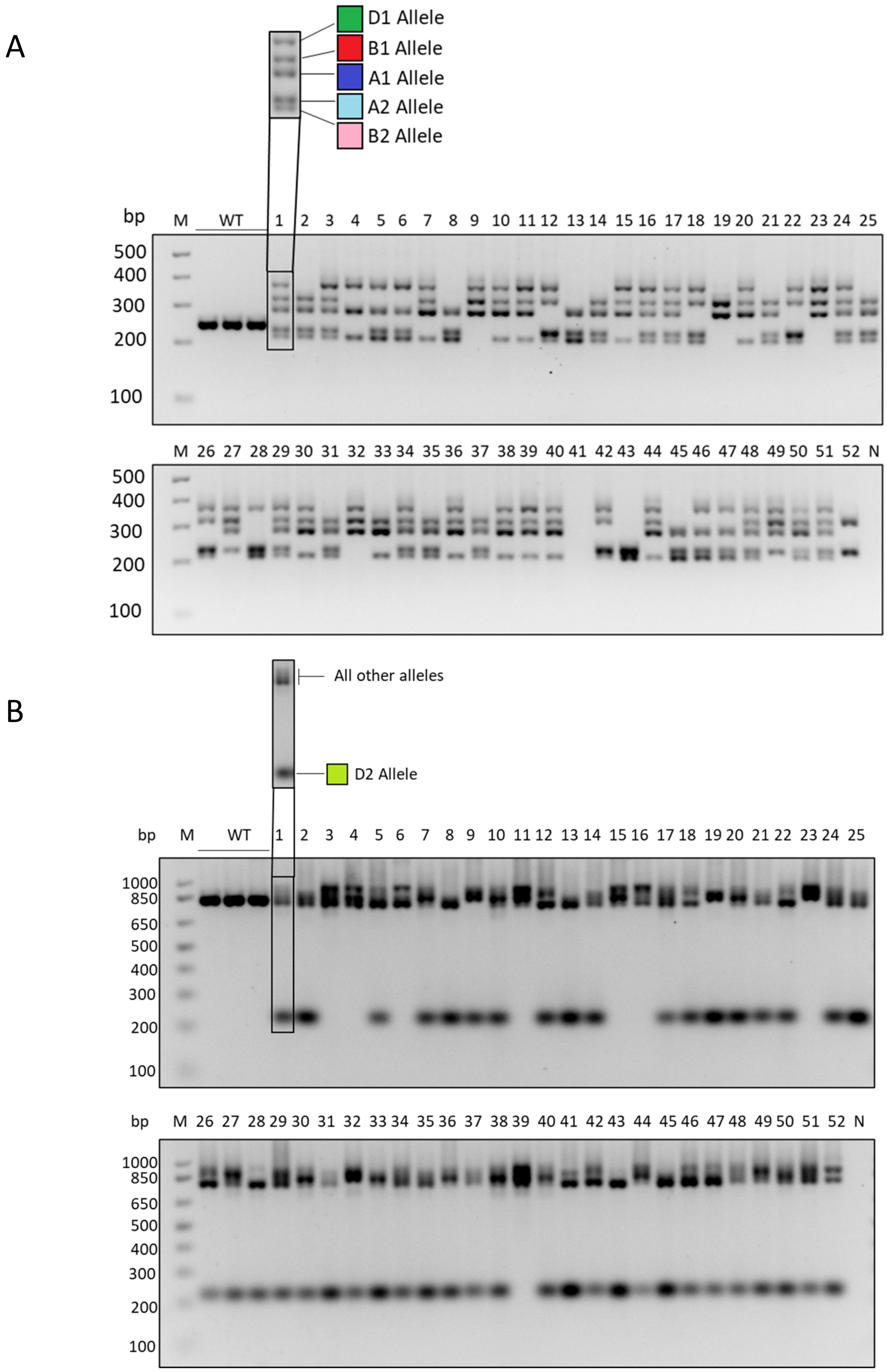

Figure S6

PCR-genotyping of T1 progeny of *tabak1-2* T0 plant 1 with FH227 + FH228 primers (A) and FH227 + FH230 primers (B).

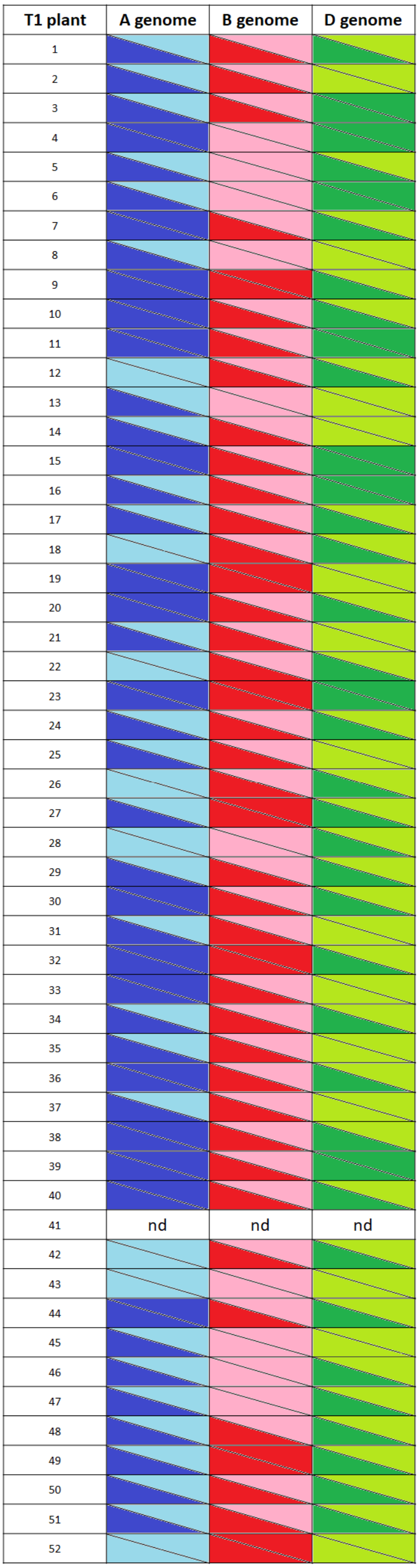

Figure S7

TaBAK1-2 allele distribution among T1 progeny of *tabak1-2* T0 plant 1.

```
TaBAK1-2_A(WT)        1 MAASPEMLRRCWWAAAAVLSAVLTVSRVSANTEGDALYSLRQSLKDANNVLQSWDPTLVN  60
                        MAASPEMLRRCWWAAAAVLSAVLTVSRVSANTEGDALYSLRQSLKDANNVLQSWDPTLVN
TaBAK1-2_T0-1_A1      1 MAASPEMLRRCWWAAAAVLSAVLTVSRVSANTEGDALYSLRQSLKDANNVLQSWDPTLVN  60

TaBAK1-2_A(WT)       61 PCTWFHVTCNTDNSVIRVDLGNAQLSGALVSQLGQLKNLQYLELYSNNISGTIPLELGN- 119
                        PCTWFHVTCNTDNSVIRVDLGNAQLSGALVSQLGQLKNLQYLELYSNNISGTIPL   +
TaBAK1-2_T0-1_A1     61 PCTWFHVTCNTDNSVIRVDLGNAQLSGALVSQLGQLKNLQYLELYSNNISGTIPLRRQS* 120

TaBAK1-2_A(WT)      120 -LTNLVSLDLYLNKFTGGIPDTLGKLLKLRFLRLNNNSLSGQIPQS------LTNISTLQ 172
                         L    +          G   ++ + +  R+ R    +L   IP S       +N + LQ
TaBAK1-2_T0-1_A1    121 VLGCRKAGG*TRELDKPGQFGSVPEQIHWRYSRHIGETLKVAIPPS*QQQSFWSNSTILQ 180

TaBAK1-2_A(WT)      173 VLDLSNNNLSGAVPSTGSFSLFTPISFGNNPNLCGPGTTKPCPGAPPFSPPPPFNPPTPP 232
                        VLDLSNNNLSGAVPSTGSFSLFTPISFGNNPNLCGPGTTKPCPGAPPFSPPPPFNPPTPP
TaBAK1-2_T0-1_A1    181 VLDLSNNNLSGAVPSTGSFSLFTPISFGNNPNLCGPGTTKPCPGAPPFSPPPPFNPPTPP 240

TaBAK1-2_A(WT)      233 AAQGDPKTGAIAGGVAAGAALIFAVPAIGFALWRRRKPEEHFFDVPAEEDPEVHLGQLKR 292
                        AAQGDPKTGAIAGGVAAGAALIFAVPAIGFALWRRRKPEEHFFDVPAEEDPEVHLGQLKR
TaBAK1-2_T0-1_A1    241 AAQGDPKTGAIAGGVAAGAALIFAVPAIGFALWRRRKPEEHFFDVPAEEDPEVHLGQLKR 300

TaBAK1-2_A(WT)      293 FSLRELQVASDNFSNKNILGRGGFGKVYKGRLTDGTLVAVKRLKEERTPGGELQFQTEVE 352
                        FSLRELQVASDNFSNKNILGRGGFGKVYKGRLTDGTLVAVKRLKEERTPGGELQFQTEVE
TaBAK1-2_T0-1_A1    301 FSLRELQVASDNFSNKNILGRGGFGKVYKGRLTDGTLVAVKRLKEERTPGGELQFQTEVE 360

TaBAK1-2_A(WT)      353 MISMAVHRNLLRLRGFCMTPTERLLVYPYMANGSVASRLRERGPNEPALEWEKRTRIALG 412
                        MISMAVHRNLLRLRGFCMTPTERLLVYPYMANGSVASRLRERGPNEPALEWEKRTRIALG
TaBAK1-2_T0-1_A1    361 MISMAVHRNLLRLRGFCMTPTERLLVYPYMANGSVASRLRERGPNEPALEWEKRTRIALG 420

TaBAK1-2_A(WT)      413 SARGLSYLHDHCDPKIIHRDVKAANILLDEDFEAVVGDFGLAKLMDYKDTHVTTAVRGTI 472
                        SARGLSYLHDHCDPKIIHRDVKAANILLDEDFEAVVGDFGLAKLMDYKDTHVTTAVRGTI
TaBAK1-2_T0-1_A1    421 SARGLSYLHDHCDPKIIHRDVKAANILLDEDFEAVVGDFGLAKLMDYKDTHVTTAVRGTI 480

TaBAK1-2_A(WT)      473 GHIAPEYLSTGKSSEKTDVFGYGIMLLELITGQRAFDLARLANDDDVMLLDWVKGLLKEK 532
                        GHIAPEYLSTGKSSEKTDVFGYGIMLLELITGQRAFDLARLANDDDVMLLDWVKGLLKEK
TaBAK1-2_T0-1_A1    481 GHIAPEYLSTGKSSEKTDVFGYGIMLLELITGQRAFDLARLANDDDVMLLDWVKGLLKEK 540

TaBAK1-2_A(WT)      533 KVEMLVDPDLQSVYVEHEVEALIQVALLCTQGSPMDRPKMSEVVRMLEGDGLAERWEEWQ 592
                        KVEMLVDPDLQSVYVEHEVEALIQVALLCTQGSPMDRPKMSEVVRMLEGDGLAERWEEWQ
TaBAK1-2_T0-1_A1    541 KVEMLVDPDLQSVYVEHEVEALIQVALLCTQGSPMDRPKMSEVVRMLEGDGLAERWEEWQ 600

TaBAK1-2_A(WT)      593 KVEVVRQEAELAPRNNDWIVDSTYNLRAVELSGPR- 627
                        KVEVVRQEAELAPRNNDWIVDSTYNLRAVELSGPR
TaBAK1-2_T0-1_A1    601 KVEVVRQEAELAPRNNDWIVDSTYNLRAVELSGPR* 636
```

Figure S8

Protein alignment of the *tabak1-2* T0 plant 1 A1 allele to the WT.

```
TaBAK1-2_A(WT)       1 MAASPEMLRRCWWAAAAVLSAVLTVSRVSANTEGDALYSLRQSLKDANNVLQSWDPTLVN  60
                         MAASPEMLRRCWWAAAAVLSAVLTVSRVSANTEGDALYSLRQSLKDANNVLQSWDPTLVN
TaBAK1-2_T0-1_A2     1 MAASPEMLRRCWWAAAAVLSAVLTVSRVSANTEGDALYSLRQSLKDANNVLQSWDPTLVN  60

TaBAK1-2_A(WT)      61 PCTWFHVTCNTDNSVIRVDLGNAQLSGALVSQLGQLKNLQYLELYSNNISGTIPLELGNL 120
                         PCTWFHVTCNTDNSVIRVDLGNAQLSGALVSQLGQLKNLQYLELYSN     I  ELGNL
TaBAK1-2_T0-1_A2    61 PCTWFHVTCNTDNSVIRVDLGNAQLSGALVSQLGQLKNLQYLELYSN----NISGELGNL 116

TaBAK1-2_A(WT)     121 TNLVSLDLYLNKFTGGIPDTLGKLLKLRFLRLNNNSLSGQIPQSLTNISTLQVLDLSNNN 180
                         TNLVSLDLYLNKFTGGIPDTLGKLLKLRFLRLNNNSLSGQIPQSLT     +VLDLSNNN
TaBAK1-2_T0-1_A2   117 TNLVSLDLYLNKFTGGIPDTLGKLLKLRFLRLNNNSLSGQIPQSLT----NKVLDLSNNN 172

TaBAK1-2_A(WT)     181 LSGAVPSTGSFSLFTPISFGNNPNLCGPGTTKPCPGAPPFSPPPPFNPPTPPAAQGDPKT 240
                         LSGAVPSTGSFSLFTPISFGNNPNLCGPGTTKPCPGAPPFSPPPPFNPPTPPAAQGDPKT
TaBAK1-2_T0-1_A2   173 LSGAVPSTGSFSLFTPISFGNNPNLCGPGTTKPCPGAPPFSPPPPFNPPTPPAAQGDPKT 232

TaBAK1-2_A(WT)     241 GAIAGGVAAGAALIFAVPAIGFALWRRRKPEEHFFDVPAEEDPEVHLGQLKRFSLRELQV 300
                         GAIAGGVAAGAALIFAVPAIGFALWRRRKPEEHFFDVPAEEDPEVHLGQLKRFSLRELQV
TaBAK1-2_T0-1_A2   233 GAIAGGVAAGAALIFAVPAIGFALWRRRKPEEHFFDVPAEEDPEVHLGQLKRFSLRELQV 292

TaBAK1-2_A(WT)     301 ASDNFSNKNILGRGGFGKVYKGRLTDGTLVAVKRLKEERTPGGELQFQTEVEMISMAVHR 360
                         ASDNFSNKNILGRGGFGKVYKGRLTDGTLVAVKRLKEERTPGGELQFQTEVEMISMAVHR
TaBAK1-2_T0-1_A2   293 ASDNFSNKNILGRGGFGKVYKGRLTDGTLVAVKRLKEERTPGGELQFQTEVEMISMAVHR 352

TaBAK1-2_A(WT)     361 NLLRLRGFCMTPTERLLVYPYMANGSVASRLRERGPNEPALEWEKRTRIALGSARGLSYL 420
                         NLLRLRGFCMTPTERLLVYPYMANGSVASRLRERGPNEPALEWEKRTRIALGSARGLSYL
TaBAK1-2_T0-1_A2   353 NLLRLRGFCMTPTERLLVYPYMANGSVASRLRERGPNEPALEWEKRTRIALGSARGLSYL 412

TaBAK1-2_A(WT)     421 HDHCDPKIIHRDVKAANILLDEDFEAVVGDFGLAKLMDYKDTHVTTAVRGTIGHIAPEYL 480
                         HDHCDPKIIHRDVKAANILLDEDFEAVVGDFGLAKLMDYKDTHVTTAVRGTIGHIAPEYL
TaBAK1-2_T0-1_A2   413 HDHCDPKIIHRDVKAANILLDEDFEAVVGDFGLAKLMDYKDTHVTTAVRGTIGHIAPEYL 472

TaBAK1-2_A(WT)     481 STGKSSEKTDVFGYGIMLLELITGQRAFDLARLANDDDVMLLDWVKGLLKEKKVEMLVDP 540
                         STGKSSEKTDVFGYGIMLLELITGQRAFDLARLANDDDVMLLDWVKGLLKEKKVEMLVDP
TaBAK1-2_T0-1_A2   473 STGKSSEKTDVFGYGIMLLELITGQRAFDLARLANDDDVMLLDWVKGLLKEKKVEMLVDP 532

TaBAK1-2_A(WT)     541 DLQSVYYVEHEVEALIQVALLCTQGSPMDRPKMSEVVRMLEGDGLAERWEEWQKVEVVRQE 600
                         DLQSVYYVEHEVEALIQVALLCTQGSPMDRPKMSEVVRMLEGDGLAERWEEWQKVEVVRQE
TaBAK1-2_T0-1_A2   533 DLQSVYYVEHEVEALIQVALLCTQGSPMDRPKMSEVVRMLEGDGLAERWEEWQKVEVVRQE 592

TaBAK1-2_A(WT)     601 AELAPRNNDWIVDSTYNLRAVELSGPR 627
                         AELAPRNNDWIVDSTYNLRAVELSGPR
TaBAK1-2_T0-1_A2   593 AELAPRNNDWIVDSTYNLRAVELSGPR 619
```

Figure S9

Protein alignment of the *tabak1-2* T0 plant 1 A2 allele to the WT.

```
TaBAK1-2_B(WT)       1 MAASPEMLRRRWWAAVAVLSVVLAVSRVAANTEGDALYSLRQSLKDANNVLQSWDPTLVN  60
                       MAASPEMLRRRWWAAVAVLSVVLAVSRVAANTEGDALYSLRQSLKDANNVLQSWDPTLVN
TaBAK1-2_T0-1_B1     1 MAASPEMLRRRWWAAVAVLSVVLAVSRVAANTEGDALYSLRQSLKDANNVLQSWDPTLVN  60

TaBAK1-2_B(WT)      61 PCTWFHVTCNTDNSVIRVDLGNAQLSGALVSQLGQLKNLQYLELYSNNISGTIPLELG-- 118
                       PCTWFHVTCNTDNSVIRVDLGNAQLSGALVSQLGQLKNLQYLELYSNNISGTIPL L
TaBAK1-2_T0-1_B1    61 PCTWFHVTCNTDNSVIRVDLGNAQLSGALVSQLGQLKNLQYLELYSNNISGTIPLVLFGA 120

TaBAK1-2_B(WT)     119 ------------------------NLTNLVSLDLYLNKFTGGIPDTLGQLLKLRFLRLNN 154
                                               NLTNLVSLDLYLNKFTGGIPDTLGQLLKLRFLRLNN
TaBAK1-2_T0-1_B1   121 HTHTTRSPPNPPVGTSASRYAALGNLTNLVSLDLYLNKFTGGIPDTLGQLLKLRFLRLNN 180

TaBAK1-2_B(WT)     155 NSLSGQIPQSLTNISTLQVLDLSNNNLSGAVPSTGSFSLFTPISFGNNPNLCGPGTSKPC 214
                       NSLSGQIPQSLTNISTLQVLDLSNNNLSGAVPSTGSFSLFTPISFGNNPNLCGPGTSKPC
TaBAK1-2_T0-1_B1   181 NSLSGQIPQSLTNISTLQVLDLSNNNLSGAVPSTGSFSLFTPISFGNNPNLCGPGTSKPC 240

TaBAK1-2_B(WT)     215 PGAPPFSPPPPFNPPTPPTTPGDPKTGAIAGGVAAGAALIFAVPAIGFALWRRRKPEEHF 274
                       PGAPPFSPPPPFNPPTPPTTPGDPKTGAIAGGVAAGAALIFAVPAIGFALWRRRKPEEHF
TaBAK1-2_T0-1_B1   241 PGAPPFSPPPPFNPPTPPTTPGDPKTGAIAGGVAAGAALIFAVPAIGFALWRRRKPEEHF 300

TaBAK1-2_B(WT)     275 FDVPAEEDPEVHLGQLKRFSLRELQVASDNFSNKNILGRGGFGKVYKGRLTDGTLVAVKR 334
                       FDVPAEEDPEVHLGQLKRFSLRELQVASDNFSNKNILGRGGFGKVYKGRLTDGTLVAVKR
TaBAK1-2_T0-1_B1   301 FDVPAEEDPEVHLGQLKRFSLRELQVASDNFSNKNILGRGGFGKVYKGRLTDGTLVAVKR 360

TaBAK1-2_B(WT)     335 LKEERTPGGELQFQTEVEMISMAVHRNLLRLRGFCMTPTERLLVYPYMANGSVASRLRER 394
                       LKEERTPGGELQFQTEVEMISMAVHRNLLRLRGFCMTPTERLLVYPYMANGSVASRLRER
TaBAK1-2_T0-1_B1   361 LKEERTPGGELQFQTEVEMISMAVHRNLLRLRGFCMTPTERLLVYPYMANGSVASRLRER 420

TaBAK1-2_B(WT)     395 GPNEPALEWEKRTRIALGSARGLSYLHDHCDPKIIHRDVKAANILLDEDFEAVVGDFGLA 454
                       GPNEPALEWEKRTRIALGSARGLSYLHDHCDPKIIHRDVKAANILLDEDFEAVVGDFGLA
TaBAK1-2_T0-1_B1   421 GPNEPALEWEKRTRIALGSARGLSYLHDHCDPKIIHRDVKAANILLDEDFEAVVGDFGLA 480

TaBAK1-2_B(WT)     455 KLMDYKDTHVTTAVRGTIGHIAPEYLSTGKSSEKTDVFGYGIMLLELITGQRAFDLARLA 514
                       KLMDYKDTHVTTAVRGTIGHIAPEYLSTGKSSEKTDVFGYGIMLLELITGQRAFDLARLA
TaBAK1-2_T0-1_B1   481 KLMDYKDTHVTTAVRGTIGHIAPEYLSTGKSSEKTDVFGYGIMLLELITGQRAFDLARLA 540

TaBAK1-2_B(WT)     515 NDDDVMLLDWVKGLLKEKKVEMLVDPDLQSVYVEHEVEALIQVALLCTQGSPMDRPKMSE 574
                       NDDDVMLLDWVKGLLKEKKVEMLVDPDLQSVYVEHEVEALIQVALLCTQGSPMDRPKMSE
TaBAK1-2_T0-1_B1   541 NDDDVMLLDWVKGLLKEKKVEMLVDPDLQSVYVEHEVEALIQVALLCTQGSPMDRPKMSE 600

TaBAK1-2_B(WT)     575 VVRMLEGDGLAERWEEWQKVEVVRQEAELAPRNNDWIVDSTYNLRAVELSGPR 627
                       VVRMLEGDGLAERWEEWQKVEVVRQEAELAPRNNDWIVDSTYNLRAVELSGPR
TaBAK1-2_T0-1_B1   601 VVRMLEGDGLAERWEEWQKVEVVRQEAELAPRNNDWIVDSTYNLRAVELSGPR 653
```

Figure S10

Protein alignment of the *tabak1-2* T0 plant 1 B1 allele to the WT.

```
TaBAK1-2_B(WT)         1 MAASPEMLRRRWWAAVAVLSVVLAVSRVAANTEGDALYSLRQSLKDANNVLQSWDPTLVN  60
                         MAASPEMLRRRWWAAVAVLSVVLAVSRVAANTEGDALYSLRQSLKDANNVLQSWDPTLVN
TaBAK1-2_T0-1_B2       1 MAASPEMLRRRWWAAVAVLSVVLAVSRVAANTEGDALYSLRQSLKDANNVLQSWDPTLVN  60

TaBAK1-2_B(WT)        61 PCTWFHVTCNTDNSVIRVDLGNAQLSGALVSQLGQLKNLQYLELYSNNISGTIPLELGNL 120
                         PCTWFHVTCNTDNSVIRVDLGNAQLSGALVSQLGQLKNLQYLELYSNNISGT
TaBAK1-2_T0-1_B2      61 PCTWFHVTCNTDNSVIRVDLGNAQLSGALVSQLGQLKNLQYLELYSNNISGTN------- 113

TaBAK1-2_B(WT)       121 TNLVSLDLYLNKFTGGIPDTLGQLLKLRFLRLNNNSLSGQIPQSLTNISTLQVLDLSNNN 180
                           LVSLDLYLNKFTGGIPDTLGQLLKLRFLRLNNNSLSGQIPQSLT  +TLQVLDLSNNN
TaBAK1-2_T0-1_B2     114 --LVSLDLYLNKFTGGIPDTLGQLLKLRFLRLNNNSLSGQIPQSLT--NTLQVLDLSNNN 169

TaBAK1-2_B(WT)       181 LSGAVPSTGSFSLFTPISFGNNPNLCGPGTSKPCPGAPPFSPPPPFNPPTPPTTPGDPKT 240
                         LSGAVPSTGSFSLFTPISFGNNPNLCGPGTSKPCPGAPPFSPPPPFNPPTPPTTPGDPKT
TaBAK1-2_T0-1_B2     170 LSGAVPSTGSFSLFTPISFGNNPNLCGPGTSKPCPGAPPFSPPPPFNPPTPPTTPGDPKT 229

TaBAK1-2_B(WT)       241 GAIAGGVAAGAALIFAVPAIGFALWRRRKPEEHFFDVPAEEDPEVHLGQLKRFSLRELQV 300
                         GAIAGGVAAGAALIFAVPAIGFALWRRRKPEEHFFDVPAEEDPEVHLGQLKRFSLRELQV
TaBAK1-2_T0-1_B2     230 GAIAGGVAAGAALIFAVPAIGFALWRRRKPEEHFFDVPAEEDPEVHLGQLKRFSLRELQV 289

TaBAK1-2_B(WT)       301 ASDNFSNKNILGRGGFGKVYKGRLTDGTLVAVKRLKEERTPGGELQFQTEVEMISMAVHR 360
                         ASDNFSNKNILGRGGFGKVYKGRLTDGTLVAVKRLKEERTPGGELQFQTEVEMISMAVHR
TaBAK1-2_T0-1_B2     290 ASDNFSNKNILGRGGFGKVYKGRLTDGTLVAVKRLKEERTPGGELQFQTEVEMISMAVHR 349

TaBAK1-2_B(WT)       361 NLLRLRGFCMTPTERLLVYPYMANGSVASRLRERGPNEPALEWEKRTRIALGSARGLSYL 420
                         NLLRLRGFCMTPTERLLVYPYMANGSVASRLRERGPNEPALEWEKRTRIALGSARGLSYL
TaBAK1-2_T0-1_B2     350 NLLRLRGFCMTPTERLLVYPYMANGSVASRLRERGPNEPALEWEKRTRIALGSARGLSYL 409

TaBAK1-2_B(WT)       421 HDHCDPKIIHRDVKAANILLDEDFEAVVGDFGLAKLMDYKDTHVTTAVRGTIGHIAPEYL 480
                         HDHCDPKIIHRDVKAANILLDEDFEAVVGDFGLAKLMDYKDTHVTTAVRGTIGHIAPEYL
TaBAK1-2_T0-1_B2     410 HDHCDPKIIHRDVKAANILLDEDFEAVVGDFGLAKLMDYKDTHVTTAVRGTIGHIAPEYL 469

TaBAK1-2_B(WT)       481 STGKSSEKTDVFGYGIMLLELITGQRAFDLARLANDDDVMLLDWVKGLLKEKKVEMLVDP 540
                         STGKSSEKTDVFGYGIMLLELITGQRAFDLARLANDDDVMLLDWVKGLLKEKKVEMLVDP
TaBAK1-2_T0-1_B2     470 STGKSSEKTDVFGYGIMLLELITGQRAFDLARLANDDDVMLLDWVKGLLKEKKVEMLVDP 529

TaBAK1-2_B(WT)       541 DLQSVYVEHEVEALIQVALLCTQGSPMDRPKMSEVVRMLEGDGLAERWEEWQKVEVVRQE 600
                         DLQSVYVEHEVEALIQVALLCTQGSPMDRPKMSEVVRMLEGDGLAERWEEWQKVEVVRQE
TaBAK1-2_T0-1_B2     530 DLQSVYVEHEVEALIQVALLCTQGSPMDRPKMSEVVRMLEGDGLAERWEEWQKVEVVRQE 589

TaBAK1-2_B(WT)       601 AELAPRNNDWIVDSTYNLRAVELSGPR 627
                         AELAPRNNDWIVDSTYNLRAVELSGPR
TaBAK1-2_T0-1_B2     590 AELAPRNNDWIVDSTYNLRAVELSGPR 616
```

Figure S11

Protein alignment of the *tabak1-2* T0 plant 1 B2 allele to the WT.

```
TaBAK1-2_D(WT)       1 MAASPEMLRRRWWAAAAVLSVVLAVSRVSANTEGDALYSLRQSLKDANNVLQSWDPTLVN  60
                       MAASPEMLRRRWWAAAAVLSVVLAVSRVSANTEGDALYSLRQSLKDANNVLQSWDPTLVN
TaBAK1-2_T0-1_D1     1 MAASPEMLRRRWWAAAAVLSVVLAVSRVSANTEGDALYSLRQSLKDANNVLQSWDPTLVN  60

TaBAK1-2_D(WT)      61 PCTWFHVTCNTDNSVIRVDLGNAQLSGALVSQLGQLKNLQYLELYSNNISGTIPLELGNL 120
                       PCTWFHVTCNTDNSVIRVDLGNAQLSGALVSQLGQLKNLQYLELYSNNISGTIPL+ G L
TaBAK1-2_T0-1_D1    61 PCTWFHVTCNTDNSVIRVDLGNAQLSGALVSQLGQLKNLQYLELYSNNISGTIPLKGGLL 120

TaBAK1-2_D(WT)     121 TNLVSLDLYLNKFTGGIPDTLGQLLKLRFLRLNNNSLSG--------------------- 159
                            L L  +K   G    L   +           L
TaBAK1-2_T0-1_D1   121 *ENRVL*LCGDKRR*GSLQRLAGNVPRLAQDH*G*RLPR*RT*QTWSVWICT*TNSLAVF 180

TaBAK1-2_D(WT)     160 ----------------------QIPQSLTNISTLQVLDLSNNNLSGAVPSTGSFSLFTPI 197
                                             +       ++  +                  F
TaBAK1-2_T0-1_D1   181 QTHWGNS*SCDSSVLTTTVFLVKFHNP*PILAPSKFWIYQTTISLERFHQLARFHSLPL* 240

TaBAK1-2_D(WT)     198 SFGNNPNLCGPGTTKPCPGAPPFSPPPPFNPPTPPAAQGDPKTGAIAGGVAAGAALIFAV 257
                                         G   F           P  +   K   +   +
TaBAK1-2_T0-1_D1   241 VLVIIQIFVARVLRNHVLGHLLFLHLHHSILQHHPRHKVTLKPEQLLEVLLRVLH*YLLF 300

TaBAK1-2_D(WT)     258 PAIGFALWRRRKPEEHFFDVPAEEDPEVHLGQLKRFSLRELQVASDNFSNKNILGRGGFG 317
                         +          +            +  L   +       ++ +   + +
TaBAK1-2_T0-1_D1   301 LQLDLHCGGDVNLKSISLMSLLRRIQKCTLAS*RGSH*GSFKLLAITSAIRTF*EEVALE 360

TaBAK1-2_D(WT)     318 KVYKGRLTDGTLVAVKRLKEERTPGGELQFQTEVEMISMAVHRNLLRLRGFCMTPTERLL 377
                       +  +G         +K  K+          + + + +
TaBAK1-2_T0-1_D1   361 RCTRGD*RMVHW*QLKD*KKNVLLVANSNSKQKSK*LVWQCIGTCFDSVDSA*HLQNGY* 420

TaBAK1-2_D(WT)     378 VYPYMANGSVASRLRERGPNEPALEWEKRTRIALGSARGLSYLHDHCDPKIIHRDVKAAN 437
                                ++      G     L  +K   +                 P+     K
TaBAK1-2_T0-1_D1   421 SIHTWLTVALHHVCESEGQMSRLLNGKKELGLHWDLPEDCLTCMITVIPRSFIVMSKPQT 480

TaBAK1-2_D(WT)     438 ILLDEDFEAVVGDFGLAKLMDYKDTHVTTAVRGTIGHIAPEYLSTGKSSEKTDVFGYGIM 497
                           +    +        L   +            G + P          +
TaBAK1-2_T0-1_D1   481 FSWMKTLRRLWVTLDWPSLWTTRTLM*PQPSVERSGILLPSTYPPESPPRRRMFSVTASC 540

TaBAK1-2_D(WT)     498 LLELITGQRAFDLARLANDDDVMLLDWVKGLLKEKKVEMLVDPDLQSVYVEHEVEALIQV 557
                          L  G+       L        L   KG  K ++         ++           +
TaBAK1-2_T0-1_D1   541 FWSLSLGRGHSTSHVLQTTTMSCCLTG*KGC*KRRRWRCWWTRTCRACTWSTRWRR*SRW 600

TaBAK1-2_D(WT)     558 ALLCTQGSPMDRPKMSEVVRMLEGDGLAERWEEWQKVEVVRQEAELAPRNNDWIVDSTYN 617
                            +G      P+          G        ++     +
TaBAK1-2_T0-1_D1   601 RCCARRGRRWTGPRCRRW*GCWRATGWRSAGRSGRRWRWSGRRRSWPRETTTGSSTPPTT 660

TaBAK1-2_D(WT)     618 LRAVELSGPR 627

TaBAK1-2_T0-1_D1   661 SGRWSSPARG 670
```

Figure S12

Protein alignment of the *tabak1-2* T0 plant 1 D1 allele to the WT.

```
TaBAK1-2_D(WT)       1 MAASPEMLRRRWWAAAAVLSVVLAVSRVSANTEGDALYSLRQSLKDANNVLQSWDPTLVN  60
                       MAASPEMLRRRWWAAAAVLSVVLAVSRVSANTEGDALYSLRQSLKDANNVLQSWDPTLVN
TaBAK1-2_T0-1_D2     1 MAASPEMLRRRWWAAAAVLSVVLAVSRVSANTEGDALYSLRQSLKDANNVLQSWDPTLVN  60

TaBAK1-2_D(WT)      61 PCTWFHVTCNTDNSVIRVDLGNAQLSGALVSQLGQLKNLQYLELYSNNISGTIPLELGNL 120
                       PCTWFHVTCNTDNSVIRVDLGNAQLSGALVSQLGQLKNLQYLELYSNNISGTIPL
TaBAK1-2_T0-1_D2    61 PCTWFHVTCNTDNSVIRVDLGNAQLSGALVSQLGQLKNLQYLELYSNNISGTIPLPSKFW 120

TaBAK1-2_D(WT)     121 TNLVSLDLYLNKFTGGIPDTLGQLLKLRFLRLNNNSLSGQIPQSLTNISTLQVLDLSNNN 180
                           ++ L               +L +  + +     +  +   L       +L     +
TaBAK1-2_T0-1_D2   121 IYQTTISLERFHQLARFHSLPL*VLVIIQIFVARVLRNHVLGHLLFLHLHHSILQHHPRH 180

TaBAK1-2_D(WT)     181 LSGAVPSTGSFSLFTPISFGNNPNLCGPGTTKPCPGAPPFSPPPPFNPPTPPAAQGDPKT 240
                            P      L   +
TaBAK1-2_T0-1_D2   181 KVTLKPEQLLEVLLRVLH*----------------------------------------- 199

TaBAK1-2_D(WT)     241 GAIAGGVAAGAALIFAVPAIGFALWRRRKPEEHFFDVPAEEDPEVHLGQLKRFSLRELQV 300
                                          +          +            +  L   +       ++
TaBAK1-2_T0-1_D2   200 -------------YLLFLQLDLHCGGDVNLKSISLMSLLRRIQKCTLAS*RGSH*GSFKL 246

TaBAK1-2_D(WT)     301 ASDNFSNKNILGRGGFGKVYKGRLTDGTLVAVKRLKEERTPGGELQFQTEVEMISMAVHR 360
                        +   + +         +  +G         +K  K+          + + + +
TaBAK1-2_T0-1_D2   247 LAITSAIRTF*EEVALERCTRGD*RMVHW*QLKD*KKNVLLVANSNSKQKSK*LVWQCIG 306

TaBAK1-2_D(WT)     361 NLLRLRGFCMTPTERLLVYPYMANGSVASRLRERGPNEPALEWEKRTRIALGSARGLSYL 420
                                                ++       G     L  +K   +
TaBAK1-2_T0-1_D2   307 TCFDSVDSA*HLQNGY*SIHTWLTVALHHVCESEGQMSRLLNGKKELGLHWDLPEDCLTC 366

TaBAK1-2_D(WT)     421 HDHCDPKIIHRDVKAANILLDEDFEAVVGDFGLAKLMDYKDTHVTTAVRGTIGHIAPEYL 480
                            P+      K       +    +        L   +            G + P
TaBAK1-2_T0-1_D2   367 MITVIPRSFIVMSKPQTFSWMKTLRRLWVTLDWPSLWTTRTLM*PQPSVERSGILLPSTY 426

TaBAK1-2_D(WT)     481 STGKSSEKTDVFGYGIMLLELITGQRAFDLARLANDDDVMLLDWVKGLLKEKKVEMLVDP 540
                              +            L  G+       L        L   KG  K ++
TaBAK1-2_T0-1_D2   427 PPESPPRRRMFSVTASCFWSLSLGRGHSTSHVLQTTTMSCCLTG*KGC*KRRRWRCWWTR 486

TaBAK1-2_D(WT)     541 DLQSVYVEHEVEALIQVALLCTQGSPMDRPKMSEVVRMLEGDGLAERWEEWQKVEVVRQE 600
                         ++           +      +G     P+           G        ++     +
TaBAK1-2_T0-1_D2   487 TCRACTWSTRWRR*SRWRCCARRGRRWTGPRCRRW*GCWRATGWRSAGRSGRRWRWSGRR 546

TaBAK1-2_D(WT)     601 AELAPRNNDWIVDSTYNLRAVELSGPR 627

TaBAK1-2_T0-1_D2   547 RSWPRETTTGSSTPPTTSGRWSSPARG 573
```

Figure S13

Protein alignment of the *tabak1-2* T0 plant 1 D2 allele to the WT.

```
TaBAK1-2_A(WT)        1 MAASPEMLRRCWWAAAAVLSAVLTVSRVSANTEGDALYSLRQSLKDANNVLQSWDPTLVN  60
                        MAASPEMLRRCWWAAAAVLSAVLTVSRVSANTEGDALYSLRQSLKDANNVLQSWDPTLVN
BAK1-2A_T0-2_A1       1 MAASPEMLRRCWWAAAAVLSAVLTVSRVSANTEGDALYSLRQSLKDANNVLQSWDPTLVN  60

TaBAK1-2_A(WT)       61 PCTWFHVTCNTDNSVIRVDLGNAQLSGALVSQLGQLKNLQYLELYSNNISGTIPLELGNL 120
                        PCTWFHVTCNTDNSVIRVDLGNAQLSGALVSQLGQLKNLQYLELYSNNISGTIPL
BAK1-2A_T0-2_A1      61 PCTWFHVTCNTDNSVIRVDLGNAQLSGALVSQLGQLKNLQYLELYSNNISGTIPLVS--- 117

TaBAK1-2_A(WT)      121 TNLVSLDLYLNKFTGGIPDTLGKLLKLRFLRLNNNSLSGQIPQSLTNISTLQVLDLSNNN 180
                                                                          TLQVLDLSNNN
BAK1-2A_T0-2_A1     118 -------------------------------------------------TLQVLDLSNNN 128

TaBAK1-2_A(WT)      181 LSGAVPSTGSFSLFTPISFGNNPNLCGPGTTKPCPGAPPFSPPPPFNPPTPPAAQGDPKT 240
                        LSGAVPSTGSFSLFTPISFGNNPNLCGPGTTKPCPGAPPFSPPPPFNPPTPPAAQGDPKT
BAK1-2A_T0-2_A1     129 LSGAVPSTGSFSLFTPISFGNNPNLCGPGTTKPCPGAPPFSPPPPFNPPTPPAAQGDPKT 188

TaBAK1-2_A(WT)      241 GAIAGGVAAGAALIFAVPAIGFALWRRRKPEEHFFDVPAEEDPEVHLGQLKRFSLRELQV 300
                        GAIAGGVAAGAALIFAVPAIGFALWRRRKPEEHFFDVPAEEDPEVHLGQLKRFSLRELQV
BAK1-2A_T0-2_A1     189 GAIAGGVAAGAALIFAVPAIGFALWRRRKPEEHFFDVPAEEDPEVHLGQLKRFSLRELQV 248

TaBAK1-2_A(WT)      301 ASDNFSNKNILGRGGFGKVYKGRLTDGTLVAVKRLKEERTPGGELQFQTEVEMISMAVHR 360
                        ASDNFSNKNILGRGGFGKVYKGRLTDGTLVAVKRLKEERTPGGELQFQTEVEMISMAVHR
BAK1-2A_T0-2_A1     249 ASDNFSNKNILGRGGFGKVYKGRLTDGTLVAVKRLKEERTPGGELQFQTEVEMISMAVHR 308

TaBAK1-2_A(WT)      361 NLLRLRGFCMTPTERLLVYPYMANGSVASRLRERGPNEPALEWEKRTRIALGSARGLSYL 420
                        NLLRLRGFCMTPTERLLVYPYMANGSVASRLRERGPNEPALEWEKRTRIALGSARGLSYL
BAK1-2A_T0-2_A1     309 NLLRLRGFCMTPTERLLVYPYMANGSVASRLRERGPNEPALEWEKRTRIALGSARGLSYL 368

TaBAK1-2_A(WT)      421 HDHCDPKIIHRDVKAANILLDEDFEAVVGDFGLAKLMDYKDTHVTTAVRGTIGHIAPEYL 480
                        HDHCDPKIIHRDVKAANILLDEDFEAVVGDFGLAKLMDYKDTHVTTAVRGTIGHIAPEYL
BAK1-2A_T0-2_A1     369 HDHCDPKIIHRDVKAANILLDEDFEAVVGDFGLAKLMDYKDTHVTTAVRGTIGHIAPEYL 428

TaBAK1-2_A(WT)      481 STGKSSEKTDVFGYGIMLLELITGQRAFDLARLANDDDVMLLDWVKGLLKEKKVEMLVDP 540
                        STGKSSEKTDVFGYGIMLLELITGQRAFDLARLANDDDVMLLDWVKGLLKEKKVEMLVDP
BAK1-2A_T0-2_A1     429 STGKSSEKTDVFGYGIMLLELITGQRAFDLARLANDDDVMLLDWVKGLLKEKKVEMLVDP 488

TaBAK1-2_A(WT)      541 DLQSVYVEHEVEALIQVALLCTQGSPMDRPKMSEVVRMLEGDGLAERWEEWQKVEVVRQE 600
                        DLQSVYVEHEVEALIQVALLCTQGSPMDRPKMSEVVRMLEGDGLAERWEEWQKVEVVRQE
BAK1-2A_T0-2_A1     489 DLQSVYVEHEVEALIQVALLCTQGSPMDRPKMSEVVRMLEGDGLAERWEEWQKVEVVRQE 548

TaBAK1-2_A(WT)      601 AELAPRNNDWIVDSTYNLRAVELSGPR 627
                        AELAPRNNDWIVDSTYNLRAVELSGPR
BAK1-2A_T0-2_A1     549 AELAPRNNDWIVDSTYNLRAVELSGPR 575
```

Figure S14

Protein alignment of the *tabak1-2* T0 plant 2 A1 allele to the WT.

```
TaBAK1-2_D(WT)       1 MAASPEMLRRRWWAAAAVLSVVLAVSRVSANTEGDALYSLRQSLKDANNVLQSWDPTLVN  60
                       MAASPEMLRRRWWAAAAVLSVVLAVSRVSANTEGDALYSLRQSLKDANNVLQSWDPTLVN
TaBAK1-2_T0-2_D1     1 MAASPEMLRRRWWAAAAVLSVVLAVSRVSANTEGDALYSLRQSLKDANNVLQSWDPTLVN  60

TaBAK1-2_D(WT)      61 PCTWFHVTCNTDNSVIRVDLGNAQLSGALVSQLGQLKNLQYLELYSNNISGTIPLELGNL 120
                       PCTWFHVTCNTDNSVIRVDLGNAQLSGALVSQLGQLKNLQYLELYSNNISGTIPL
TaBAK1-2_T0-2_D1    61 PCTWFHVTCNTDNSVIRVDLGNAQLSGALVSQLGQLKNLQYLELYSNNISGTIPLSG-T* 119

TaBAK1-2_D(WT)     121 TNLVSLDLYLNKFTGGIPDTLGQLLKLRFLRLNNNSLSGQIPQSLTNISTLQVLDLSNNN 180
                                            G         L       +       ++  +
TaBAK1-2_T0-2_D1   120 QTWSVWICT*TNSLAVFQTHWGNS*SCDSSVLTTTVFLVKFHNP*PILAPSKFWIYQTTI 179

TaBAK1-2_D(WT)     181 LSGAVPSTGSFSLFTPISFGNNPNLCGPGTTKPCPGAPPFSPPPPFNPPTPPAAQGDPKT 240
                                 F                        G   F           P  +   K
TaBAK1-2_T0-2_D1   180 SLERFHQLARFHSLPL*VLVIIQIFVARVLRNHVLGHLLFLHLHHSILQHHPRHKVTLKP 239

TaBAK1-2_D(WT)     241 GAIAGGVAAGAALIFAVPAIGFALWRRRKPEEHFFDVPAEEDPEVHLGQLKRFSLRELQV 300
                         +   +            +          +            +  L   +       ++
TaBAK1-2_T0-2_D1   240 EQLLEVLLRVLH*YLLFLQLDLHCGGDVNLKSISLMSLLRRIQKCTLAS*RGSH*GSFKL 299

TaBAK1-2_D(WT)     301 ASDNFSNKNILGRGGFGKVYKGRLTDGTLVAVKRLKEERTPGGELQFQTEVEMISMAVHR 360
                        +   + +         +  +G         +K  K+          + + + +
TaBAK1-2_T0-2_D1   300 LAITSAIRTF*EEVALERCTRGD*RMVHW*QLKD*KKNVLLVANSNSKQKSK*LVWQCIG 359

TaBAK1-2_D(WT)     361 NLLRLRGFCMTPTERLLVYPYMANGSVASRLRERGPNEPALEWEKRTRIALGSARGLSYL 420
                                                ++       G     L  +K   +
TaBAK1-2_T0-2_D1   360 TCFDSVDSA*HLQNGY*SIHTWLTVALHHVCESEGQMSRLLNGKKELGLHWDLPEDCLTC 419

TaBAK1-2_D(WT)     421 HDHCDPKIIHRDVKAANILLDEDFEAVVGDFGLAKLMDYKDTHVTTAVRGTIGHIAPEYL 480
                            P+      K       +    +        L   +            G + P
TaBAK1-2_T0-2_D1   420 MITVIPRSFIVMSKPQTFSWMKTLRRLWVTLDWPSLWTTRTLM*PQPSVERSGILLPSTY 479

TaBAK1-2_D(WT)     481 STGKSSEKTDVFGYGIMLLELITGQRAFDLARLANDDDVMLLDWVKGLLKEKKVEMLVDP 540
                              +            L  G+       L        L   KG  K ++
TaBAK1-2_T0-2_D1   480 PPESPPRRRMFSVTASCFWSLSLGRGHSTSHVLQTTTMSCCLTG*KGC*KRRRWRCWWTR 539

TaBAK1-2_D(WT)     541 DLQSVYVEHEVEALIQVALLCTQGSPMDRPKMSEVVRMLEGDGLAERWEEWQKVEVVRQE 600
                         ++           +      +G     P+           G        ++     +
TaBAK1-2_T0-2_D1   540 TCRACTWSTRWRR*SRWRCCARRGRRWTGPRCRRW*GCWRATGWRSAGRSGRRWRWSGRR 599

TaBAK1-2_D(WT)     601 AELAPRNNDWIVDSTYNLRAVELSGPR 627

TaBAK1-2_T0-2_D1   600 RSWPRETTTGSSTPPTTSGRWSSPARG 626
```

Figure S15

Protein alignment of the *tabak1-2* T0 plant 2 D1 allele to the WT.

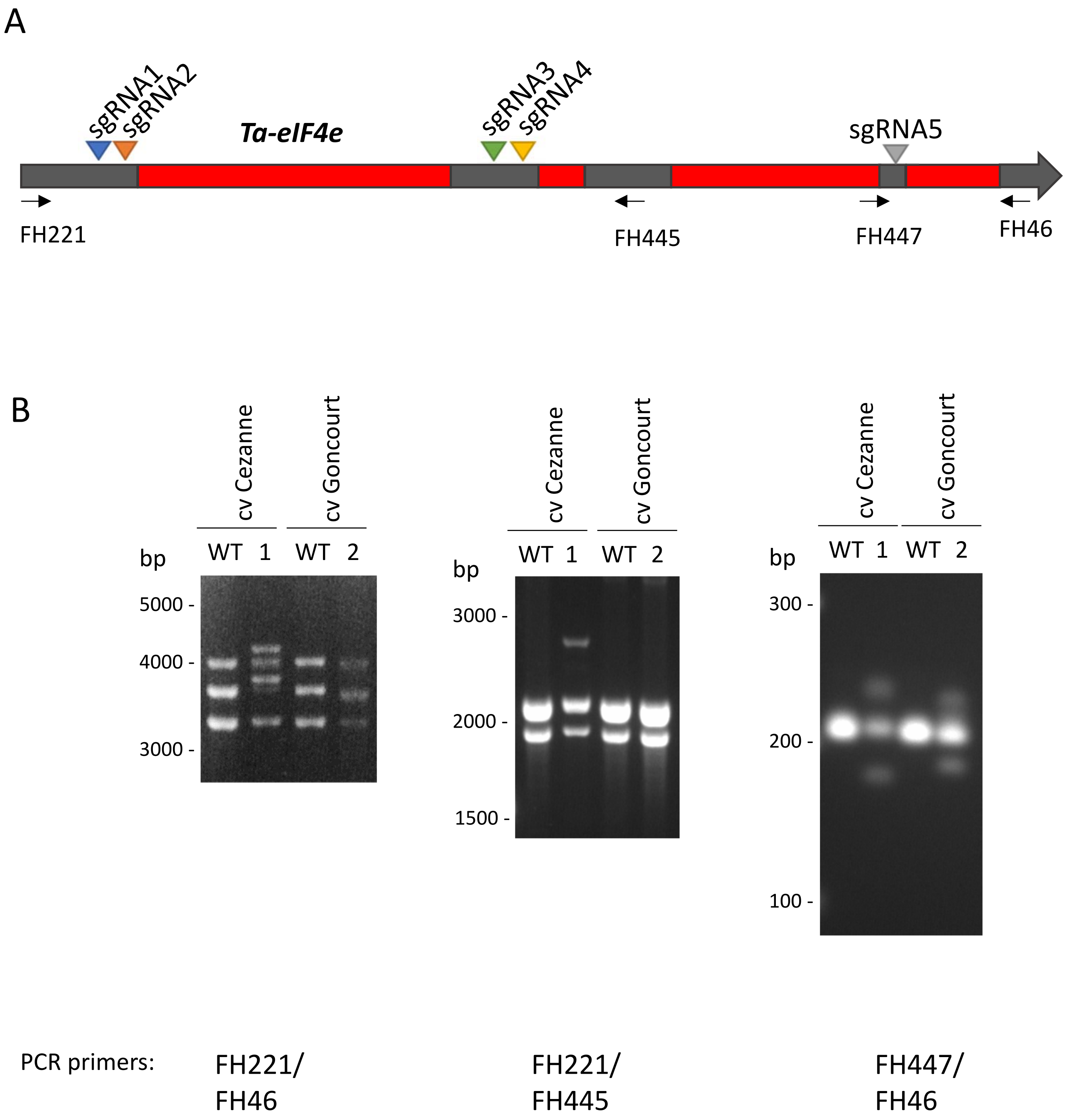

Figure S16

PCR genotyping of the *ta-eif4e* T0 plants 1 and 2 (cvs Cezanne and Goncourt, respectively). (A) A cartoon showing positions of sgRNA target sites and PCR primer binding sites within the *Ta-eIF4e* gene. Exons are shown in grey, while introns – in red. (B) Results of PCR genotyping with three primer sets.

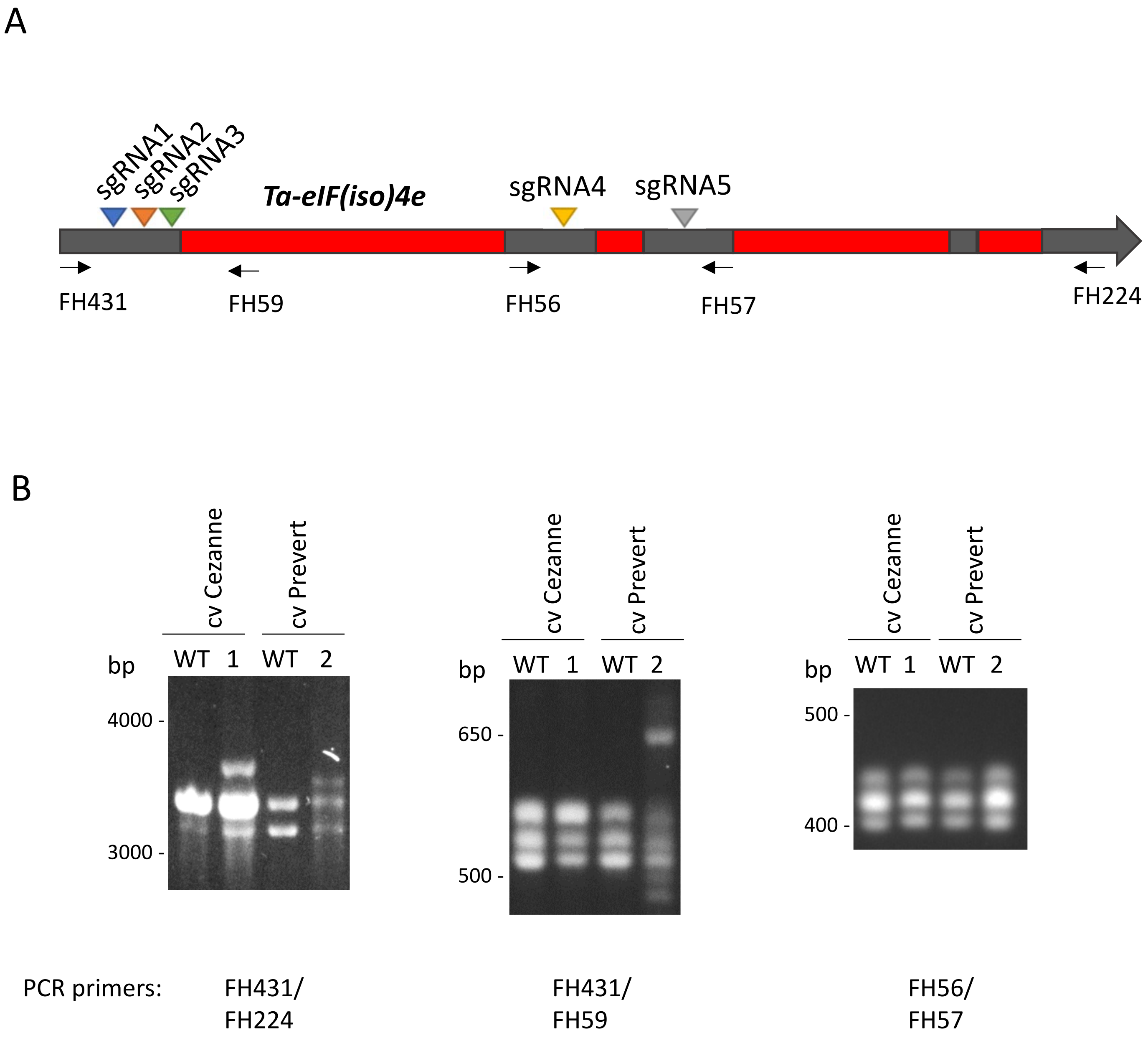

Figure S17

PCR genotyping of the *ta-eif(iso)4e* T0 plants 1 and 2 (cvs Cezanne and Prevert, respectively). (A) A cartoon showing positions of sgRNA target sites and PCR primer binding sites within the *Ta-eIF(iso)4e* gene. Exons are shown in grey, while introns – in red. (B) Results of PCR genotyping with three primer sets.

ta-eif4e

T0 Plant 1 (cv Cezanne)

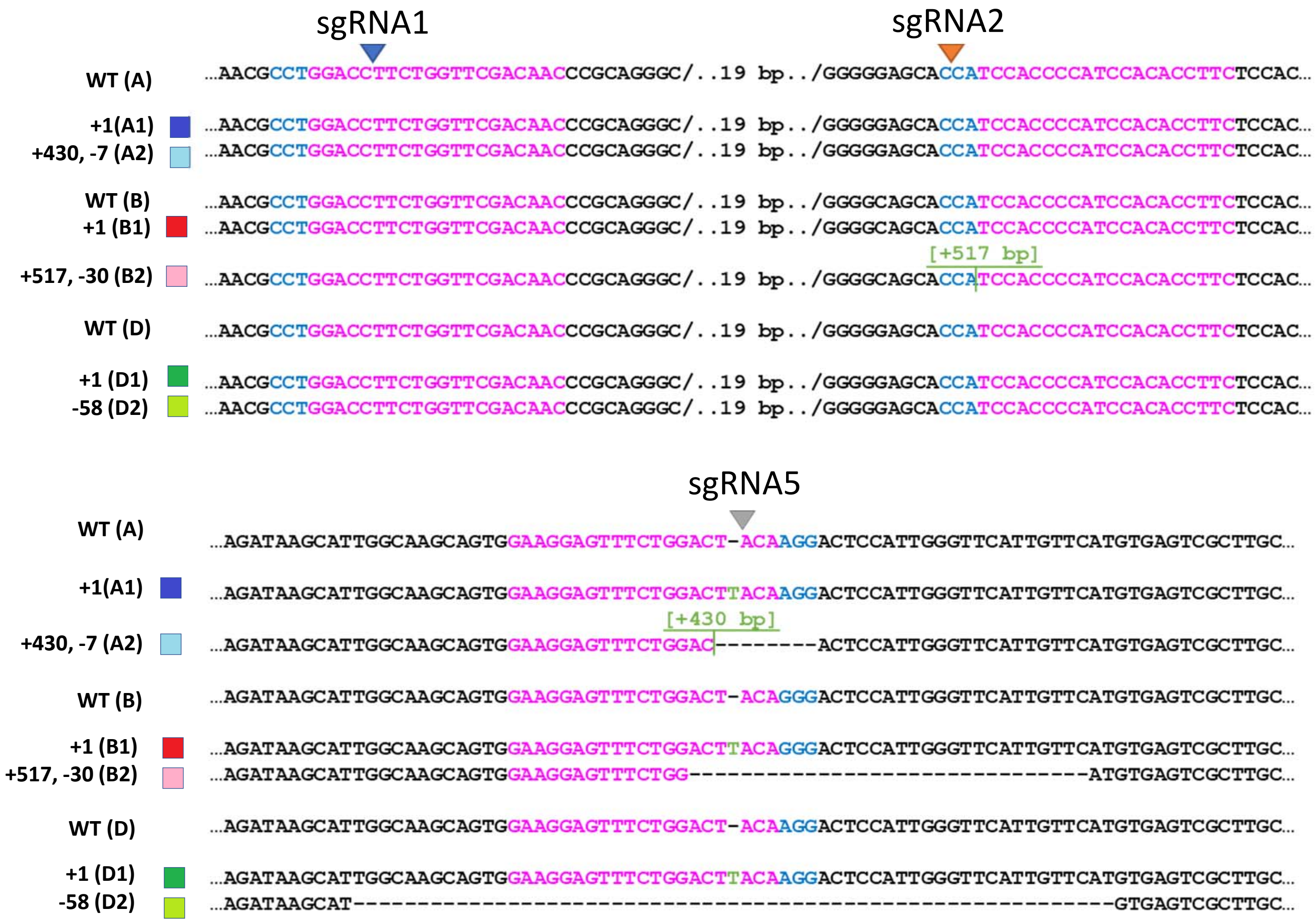

T0 Plant 2 (cv Goncourt)

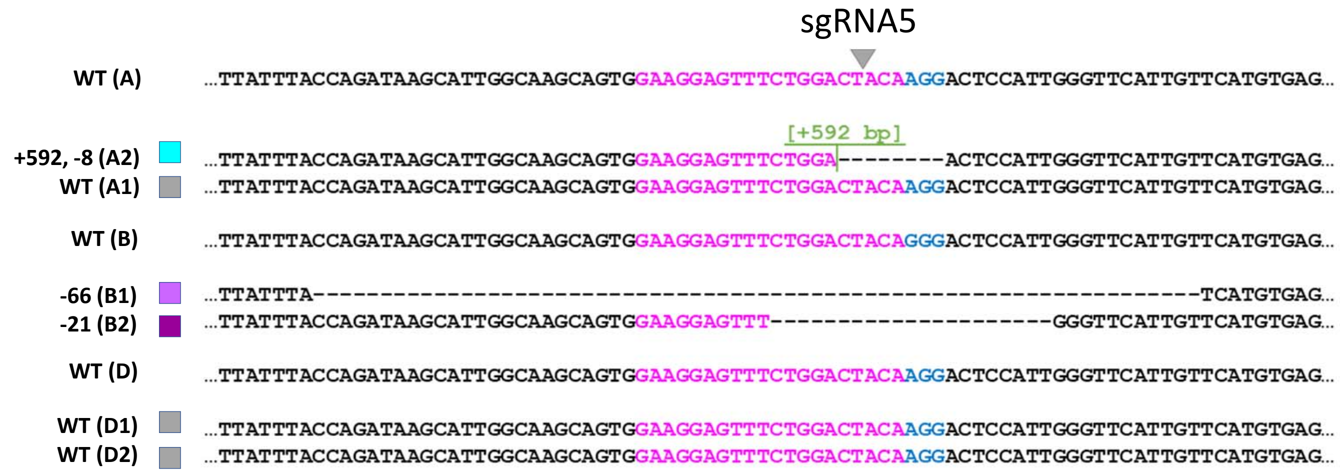

Figure S18
Alignments showing CRISPR/Cas-induced indels in *Ta-eIF4e* homoeologues in two *ta-eif4e* T0 plants (cvs Cezanne and Goncourt).

A

```
Ta-eIF4e_A(WT)       1 MAEDTETRPASAGAEEREEGEIADDGDGSSAAAAGRITAHPLENAWTFWFDNPQGKSRQV  60
                       MAEDTETRPASAGAEEREEGEIADDGDGSSAAAAGRITAHPLENAWTFWFDNPQGKSRQV
Ta-eIF4e_T0-1_A1     1 MAEDTETRPASAGAEEREEGEIADDGDGSSAAAAGRITAHPLENAWTFWFDNPQGKSRQV  60

Ta-eIF4e_A(WT)      61 AWGSTIHPIHTFSTVEDFWGLYNNIHNPSKLNVGADFHCFKNKIEPKWEDPICANGGKWT 120
                       AWGSTIHPIHTFSTVEDFWGLYNNIHNPSKLNVGADFHCFKNKIEPKWEDPICANGGKWT
Ta-eIF4e_T0-1_A1    61 AWGSTIHPIHTFSTVEDFWGLYNNIHNPSKLNVGADFHCFKNKIEPKWEDPICANGGKWT 120

Ta-eIF4e_A(WT)     121 ISCGRGKSDTFWLHTLLAMIGEQFDFGDEICGAVVSVRQKQERVAIWTKNAANEAAQISI 180
                       ISCGRGKSDTFWLHTLLAMIGEQFDFGDEICGAVVSVRQKQERVAIWTKNAANEAAQISI
Ta-eIF4e_T0-1_A1   121 ISCGRGKSDTFWLHTLLAMIGEQFDFGDEICGAVVSVRQKQERVAIWTKNAANEAAQISI 180

Ta-eIF4e_A(WT)     181 GKQWKEFLDYKDSIGFIVHEDAKRSDKGPKNRYTV* 216
                       GKQWKEFLD +           K   + P+
Ta-eIF4e_T0-1_A1   181 GKQWKEFLDLQGLHWVHCS*GCKEV*QRPQEPLHRL 216
```

B

```
Ta-eIF4e_A(WT)       1 MAEDTETRPASAGAEEREEGEIADDGDGSSAAAAGRITAHPLENAWTFWFDNPQGKSRQV  60
                       MAEDTETRPASAGAEEREEGEIADDGDGSSAAAAGRITAHPLENAWTFWFDNPQGKSRQV
Ta-eIF4e_T0-1_A2     1 MAEDTETRPASAGAEEREEGEIADDGDGSSAAAAGRITAHPLENAWTFWFDNPQGKSRQV  60

Ta-eIF4e_A(WT)      61 AWGSTIHPIHTFSTVEDFWGLYNNIHNPSKLNVGADFHCFKNKIEPKWEDPICANGGKWT 120
                       AWGSTIHPIHTFSTVEDFWGLYNNIHNPSKLNVGADFHCFKNKIEPKWEDPICANGGKWT
Ta-eIF4e_T0-1_A2    61 AWGSTIHPIHTFSTVEDFWGLYNNIHNPSKLNVGADFHCFKNKIEPKWEDPICANGGKWT 120

Ta-eIF4e_A(WT)     121 ISCGRGKSDTFWLHTLLAMIGEQFDFGDEICGAVVSVRQKQERVAIWTKNAANEAAQISI 180
                       ISCGRGKSDTFWLHTLLAMIGEQFDFGDEICGAVVSVRQKQERVAIWTKNAANEAAQ  +
Ta-eIF4e_T0-1_A2   121 ISCGRGKSDTFWLHTLLAMIGEQFDFGDEICGAVVSVRQKQERVAIWTKNAANEAAQTPL 180

Ta-eIF4e_A(WT)     181 GKQWKEFLDYKDSIGFIVHEDAKRSDKGPKNRYTV* 216
                       G  +                         ++ +
Ta-eIF4e_T0-1_A2   181 GSLFMRMQRGLTKAPRTATPFE*ALASSGRSFW--- 213
```

Figure S19
Protein alignments of the *ta-eif4e* T0 plant 1 A1 (A) and A2 (B) alleles to the WT.

A

```
Ta-eIF4e_B(WT)       1 MAEDTETRPASAGAEEREEGEIADDGDGSSAAAAGRISAHPLENAWTFWFDNPQGKSRQV  60
                       MAEDTETRPASAGAEEREEGEIADDGDGSSAAAAGRISAHPLENAWTFWFDNPQGKSRQV
Ta-eIF4e_T0-1_B1     1 MAEDTETRPASAGAEEREEGEIADDGDGSSAAAAGRISAHPLENAWTFWFDNPQGKSRQV  60

Ta-eIF4e_B(WT)      61 AWGSTIHPIHTFSTVEDFWGLYNNIHNPSKLNVGADFHCFKNKIEPKWEDPICANGGKWT 120
                       AWGSTIHPIHTFSTVEDFWGLYNNIHNPSKLNVGADFHCFKNKIEPKWEDPICANGGKWT
Ta-eIF4e_T0-1_B1    61 AWGSTIHPIHTFSTVEDFWGLYNNIHNPSKLNVGADFHCFKNKIEPKWEDPICANGGKWT 120

Ta-eIF4e_B(WT)     121 ISCGRGKSDTFWLHTLLAMIGEQFDFGDEICGAVVSVRQKQERVAIWTKNAANEAAQISI 180
                       ISCGRGKSDTFWLHTLLAMIGEQFDFGDEICGAVVSVRQKQERVAIWTKNAANEAAQISI
Ta-eIF4e_T0-1_B1   121 ISCGRGKSDTFWLHTLLAMIGEQFDFGDEICGAVVSVRQKQERVAIWTKNAANEAAQISI 180

Ta-eIF4e_B(WT)     181 GKQWKEFLDYRDSIGFIVHEDAKRSDKGPKNRYTV* 216
                       GKQWKEFLD +           K   + P+
Ta-eIF4e_T0-1_B1   181 GKQWKEFLDLQGLHWVHCS*GCKEV*QRPQEPLHRL 216
```

B

```
Ta-eIF4e_B(WT)       1 MAEDTETRPASAGAEEREEGEIADDGDGSSAAAAGRISAHPLENAWTFWFDNPQGKSRQV  60
                       MAEDTETRPASAGAEEREEGEIADDGDGSSAAAAGRISAHPLENAWTFWFDNPQGKSRQV
Ta-eIF4e_T0-1_B2     1 MAEDTETRPASAGAEEREEGEIADDGDGSSAAAAGRISAHPLENAWTFWFDNPQGKSRQV  60

Ta-eIF4e_B(WT)      61 AWGSTIHPIHTFSTVEDFWGLYNNIHNPSKLNVGADFHCFKNKIEPKWEDPICANGGKWT 120
                       AWGSTI      S       L +           A F      +E             +
Ta-eIF4e_T0-1_B2    61 AWGSTIQW*TGQSGPCRLASLASCPATAQYRTRKAAFP*SAAYLEVTIVVHDDEYFTVYN 120

Ta-eIF4e_B(WT)     121 ISCGRGKSDTFWLHTLLAMIGEQFDFGDEICGAVVSVRQKQERVAIWTKNAANEAAQISI 180
                                            ++    D +      V  + +++     +A
Ta-eIF4e_T0-1_B2   121 ELTKVKYVTEGMRKPAFLSGEQKKAIVDLLFKTNRKVTDETKKIMKSIPSAERLEPSQDS 180

Ta-eIF4e_B(WT)     181 GKQWKEFLDYRDSIGFIVHEDAKRSDKGPKNRYTV*------------------------ 216
                        K+    L  R  + +  H     S
Ta-eIF4e_T0-1_B2   181 PKRNTGKLAIRTCLTYRSHPCTNASSTDLTQTRI*HKHEQK*NYRALIMDRNADLEKVEP 240

Ta-eIF4e_B(WT)         ------------------------------------------------------------

Ta-eIF4e_T0-1_B2   241 HPHLLHRRGLLGHLLHRRGLLGPLQQYP*P*QVECGSRLPLLQE*D*A*MGRPHLCQWR* 300

Ta-eIF4e_B(WT)         ------------------------------------------------------------

Ta-eIF4e_T0-1_B2   301 MDHQLWQREI*HILVAYFAGNDW*TIRLW*RNLWSSR*RASETGKSSYLD*ECCQ*SCSD 360

Ta-eIF4e_B(WT)         ----------------------------

Ta-eIF4e_T0-1_B2   361 KHWQAVEGVSG*GCKEV*QRPQEPLHRL 388
```

Figure S20
Protein alignments of the *ta-eif4e* T0 plant 1 B1 (A) and B2 (B) alleles to the WT.

A

```
Ta-eIF4e_D(WT)        1 MAEDTETRPASAGAEEREEGEIADDGDGSSAAAAGRITAHPLENAWTFWFDNPQGKSRQV  60
                        MAEDTETRPASAGAEEREEGEIADDGDGSSAAAAGRITAHPLENAWTFWFDNPQGKSRQV
Ta-eIF4e_T0-1_D1      1 MAEDTETRPASAGAEEREEGEIADDGDGSSAAAAGRITAHPLENAWTFWFDNPQGKSRQV  60

Ta-eIF4e_D(WT)       61 AWGSTIHPIHTFSTVEDFWGLYNNIHNPSKLNVGADFHCFKNKIEPKWEDPICANGGKWT 120
                        AWGSTIHPIHTFSTVEDFWGLYNNIHNPSKLNVGADFHCFKNKIEPKWEDPICANGGKWT
Ta-eIF4e_T0-1_D1     61 AWGSTIHPIHTFSTVEDFWGLYNNIHNPSKLNVGADFHCFKNKIEPKWEDPICANGGKWT 120

Ta-eIF4e_D(WT)      121 ISCGRGKSDTFWLHTLLAMIGEQFDFGDEICGAVVSVRQKQERVAIWTKNAANEAAQISI 180
                        ISCGRGKSDTFWLHTLLAMIGEQFDFGDEICGAVVSVRQKQERVAIWTKNAANEAAQISI
Ta-eIF4e_T0-1_D1    121 ISCGRGKSDTFWLHTLLAMIGEQFDFGDEICGAVVSVRQKQERVAIWTKNAANEAAQISI 180

Ta-eIF4e_D(WT)      181 GKQWKEFLDYKDSIGFIVHEDAKRSDKGPKNRYTV* 216
                        GKQWKEFLD +           K   + P+
Ta-eIF4e_T0-1_D1    181 GKQWKEFLDLQGLHWVHCS*GCKEV*QRPQEPLHRL 216
```

B

```
Ta-eIF4e_D(WT)        1 MAEDTETRPASAGAEEREEGEIADDGDGSSAAAAGRITAHPLENAWTFWFDNPQGKSRQV  60
                        MAEDTETRPASAGAEEREEGEIADDGDGSSAAAAGRITAHPLENAWTFWFDNPQGKSRQV
Ta-eIF4e_T0-1_D2      1 MAEDTETRPASAGAEEREEGEIADDGDGSSAAAAGRITAHPLENAWTFWFDNPQGKSRQV  60

Ta-eIF4e_D(WT)       61 AWGSTIHPIHTFSTVEDFWGLYNNIHNPSKLNVGADFHCFKNKIEPKWEDPICANGGKWT 120
                        AWGSTIHPIHTFSTVEDFWGLYNNIHNPSKLNVGADFHCFKNKIEPKWEDPICANGGKWT
Ta-eIF4e_T0-1_D2     61 AWGSTIHPIHTFSTVEDFWGLYNNIHNPSKLNVGADFHCFKNKIEPKWEDPICANGGKWT 120

Ta-eIF4e_D(WT)      121 ISCGRGKSDTFWLHTLLAMIGEQFDFGDEICGAVVSVRQKQERVAIWTKNAANEAAQISI 180
                        ISCGRGKSDTFWLHTLLAMIGEQFDFGDEICGAVVSVRQKQERVAIWTKNAANEAAQIS+
Ta-eIF4e_T0-1_D2    121 ISCGRGKSDTFWLHTLLAMIGEQFDFGDEICGAVVSVRQKQERVAIWTKNAANEAAQISM 180

Ta-eIF4e_D(WT)      181 GKQWKEFLDYKDSIGFIVHEDAKRSDKGPKNRYTV* 216
                          Q       + +  F
Ta-eIF4e_T0-1_D2    181 RMQRGLTKAPRTATPF-------------------- 196
```

Figure S21

Protein alignments of the *ta-eif4e* T0 plant 1 D1 (A) and D2 (B) alleles to the WT.

```
Ta-eIF4e_A(WT)        1 MAEDTETRPASAGAEEREEGEIADDGDGSSAAAAGRITAHPLENAWTFWFDNPQGKSRQV  60
                        MAEDTETRPASAGAEEREEGEIADDGDGSSAAAAGRITAHPLENAWTFWFDNPQGKSRQV
Ta-eIF4e_T0-2_A2      1 MAEDTETRPASAGAEEREEGEIADDGDGSSAAAAGRITAHPLENAWTFWFDNPQGKSRQV  60

Ta-eIF4e_A(WT)       61 AWGSTIHPIHTFSTVEDFWGLYNNIHNPSKLNVGADFHCFKNKIEPKWEDPICANGGKWT 120
                        AWGSTIHPIHTFSTVEDFWGLYNNIHNPSKLNVGADFHCFKNKIEPKWEDPICANGGKWT
Ta-eIF4e_T0-2_A2     61 AWGSTIHPIHTFSTVEDFWGLYNNIHNPSKLNVGADFHCFKNKIEPKWEDPICANGGKWT 120

Ta-eIF4e_A(WT)      121 ISCGRGKSDTFWLHTLLAMIGEQFDFGDEICGAVVSVRQKQERVAIWTKNAANEAAQISI 180
                        ISCGRGKSDTFWLHTLLAMIGEQFDFGDEICGAVVSVRQKQERVAIWTKNAANEAAQISI
Ta-eIF4e_T0-2_A2    121 ISCGRGKSDTFWLHTLLAMIGEQFDFGDEICGAVVSVRQKQERVAIWTKNAANEAAQISI 180

Ta-eIF4e_A(WT)      181 GKQWKEFLDYKDSIGFIVHEDAKRSDKGPKNRYTV*------------------------ 216
                        GKQWKEFL+ +     +    +  S +    ++  *
Ta-eIF4e_T0-2_A2    181 GKQWKEFLELQHWGQMVSPPVS*LSTRRGVRQHKP*TLNPKP*TLTLNPKP*TLNPKP*T 240

Ta-eIF4e_A(WT)          ------------------------------------------------------------

Ta-eIF4e_T0-2_A2    241 LNPKP*TLKP*QP*TLNPKP*TLNLKP*TLNPNPNPKP*TLNPKP*TLNPNPKP*TLNPK 300

Ta-eIF4e_A(WT)          ------------------------------------------------------------

Ta-eIF4e_T0-2_A2    301 P*TQP*TLNPKP*TLNPKP*TLTLNPKPQTLNPNPMAAQ*HSCRYLRASHDRH*SGYLAD 360

Ta-eIF4e_A(WT)          --------------------------------------------------

Ta-eIF4e_T0-2_A2    361 KSKRT*RCLGRSSGGGTL*SGS*TGSTPLGSLFMRMQRGLTKAPRTATPF 410
```

Figure S22

Protein alignment of the *ta-eif4e* T0 plant 2 A2 allele to the WT.

A

```
Ta-eIF4e_B(WT)       1 MAEDTETRPASAGAEEREEGEIADDGDGSSAAAAGRISAHPLENAWTFWFDNPQGKSRQV  60
                       MAEDTETRPASAGAEEREEGEIADDGDGSSAAAAGRISAHPLENAWTFWFDNPQGKSRQV
Ta-eIF4e_T0-2_B1     1 MAEDTETRPASAGAEEREEGEIADDGDGSSAAAAGRISAHPLENAWTFWFDNPQGKSRQV  60

Ta-eIF4e_B(WT)      61 AWGSTIHPIHTFSTVEDFWGLYNNIHNPSKLNVGADFHCFKNKIEPKWEDPICANGGKWT 120
                       AWGSTIHPIHTFSTVEDFWGLYNNIHNPSKLNVGADFHCFKNKIEPKWEDPICANGGKWT
Ta-eIF4e_T0-2_B1    61 AWGSTIHPIHTFSTVEDFWGLYNNIHNPSKLNVGADFHCFKNKIEPKWEDPICANGGKWT 120

Ta-eIF4e_B(WT)     121 ISCGRGKSDTFWLHTLLAMIGEQFDFGDEICGAVVSVRQKQERVAIWTKNAANEAAQISI 180
                       ISCGRGKSDTFWLHTLLAMIGEQFDFGDEICGAVVSVRQKQERVAIWTKNAANEAAQ
Ta-eIF4e_T0-2_B1   121 ISCGRGKSDTFWLHTLLAMIGEQFDFGDEICGAVVSVRQKQERVAIWTKNAANEAAQS*G 180

Ta-eIF4e_B(WT)     181 GKQWKEFLDYRDSIGFIVHEDAKRSDKGPKNRYTV* 216
                        K+  +
Ta-eIF4e_T0-2_B1   181 CKEV*QRPQEPLHRL--------------------- 195
```

B

```
Ta-eIF4e_B(WT)       1 MAEDTETRPASAGAEEREEGEIADDGDGSSAAAAGRISAHPLENAWTFWFDNPQGKSRQV  60
                       MAEDTETRPASAGAEEREEGEIADDGDGSSAAAAGRISAHPLENAWTFWFDNPQGKSRQV
Ta-eIF4e_T0-2_B2     1 MAEDTETRPASAGAEEREEGEIADDGDGSSAAAAGRISAHPLENAWTFWFDNPQGKSRQV  60

Ta-eIF4e_B(WT)      61 AWGSTIHPIHTFSTVEDFWGLYNNIHNPSKLNVGADFHCFKNKIEPKWEDPICANGGKWT 120
                       AWGSTIHPIHTFSTVEDFWGLYNNIHNPSKLNVGADFHCFKNKIEPKWEDPICANGGKWT
Ta-eIF4e_T0-2_B2    61 AWGSTIHPIHTFSTVEDFWGLYNNIHNPSKLNVGADFHCFKNKIEPKWEDPICANGGKWT 120

Ta-eIF4e_B(WT)     121 ISCGRGKSDTFWLHTLLAMIGEQFDFGDEICGAVVSVRQKQERVAIWTKNAANEAAQISI 180
                       ISCGRGKSDTFWLHTLLAMIGEQFDFGDEICGAVVSVRQKQERVAIWTKNAANEAAQISI
Ta-eIF4e_T0-2_B2   121 ISCGRGKSDTFWLHTLLAMIGEQFDFGDEICGAVVSVRQKQERVAIWTKNAANEAAQISI 180

Ta-eIF4e_B(WT)     181 GKQWKEFLDYRDSIGFIVHEDAKRSDKGPKNRYTV* 216
                       GKQWKEF        FIVHEDAKRSDKGPKNRYTV
Ta-eIF4e_T0-2_B2   181 GKQWKEFG-------FIVHEDAKRSDKGPKNRYTV- 208
```

Figure S23
Protein alignments of the *ta-eif4e* T0 plant 2 B1 (A) and B2 (B) alleles to the WT.

ta-eif(iso)4e

T0 Plant 1 (cv Cezanne)

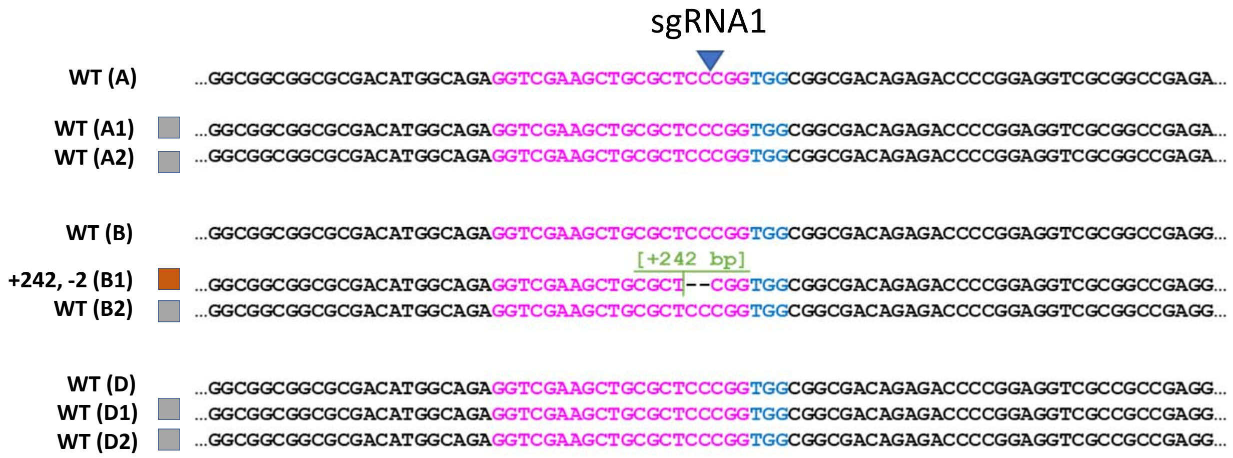

Figure S24

Alignment showing CRISPR/Cas-induced indels in *Ta-eIF(iso)4e* homoeologues in the *ta-eif(iso)4e* T0 plant 1 (cv Cezanne).

ta-eIf(iso)4e

T0 Plant 2 (cv Prevert)

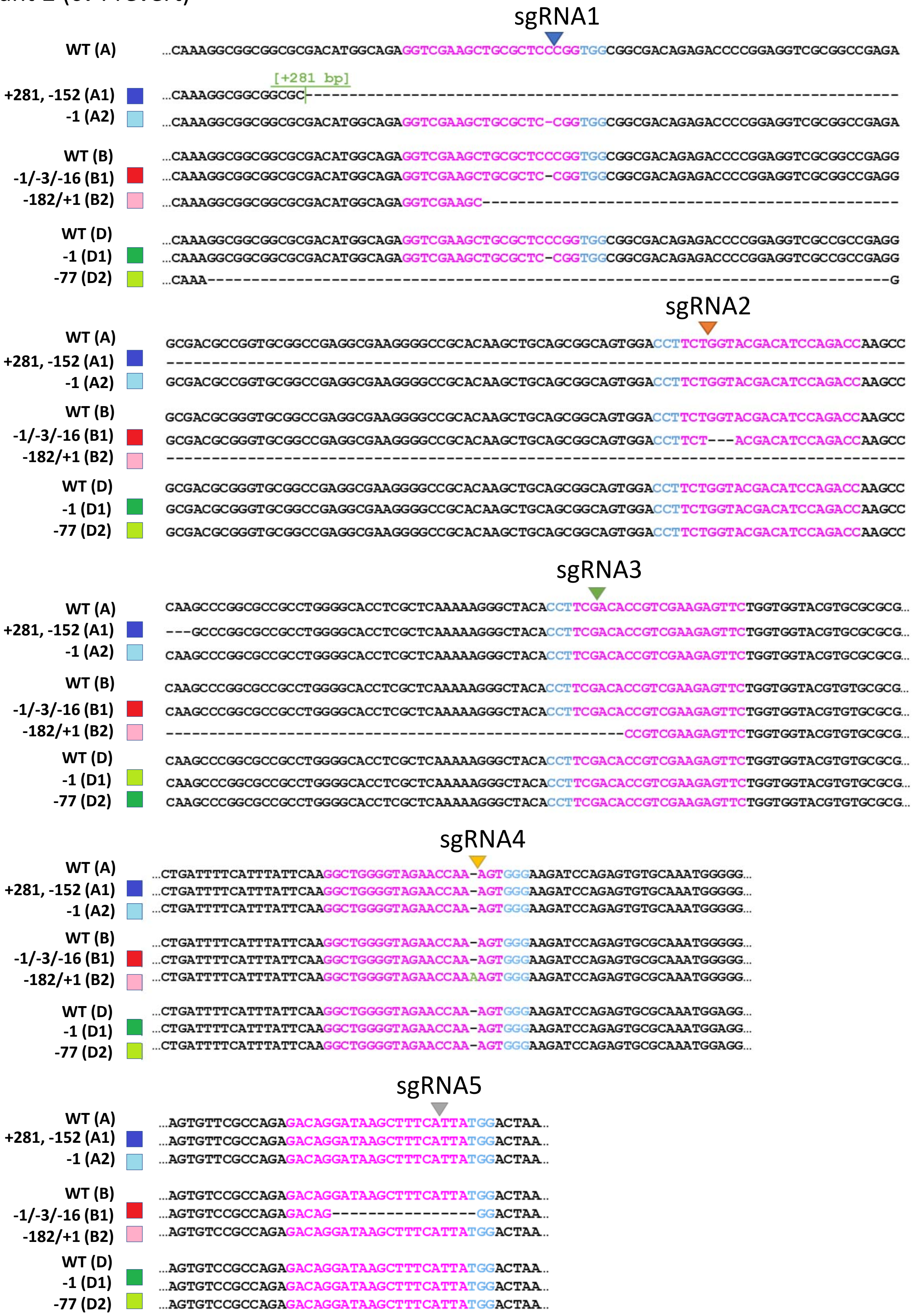

Figure S25

Alignment showing CRISPR/Cas-induced indels in *Ta-eIF(iso)4e* homoeologues in the *ta-eif(iso)4e* T0 plant 2 (cv Prevert).

```
Ta-eIF(iso)4e_B(WT)       1 MAEVEAAL--------------------P------------------------------   9
                            MAEVEAAL                    P
Ta-eIF(iso)4e_T0-1_B1     1 MAEVEAALSPVPVTSSSLLSRRRADETLPQHSAGS*VRGTSLS*TCADHGGAVLFFLFH*  60

Ta-eIF(iso)4e_B(WT)      10 -----------------------------VAATETPEVAAEGDAGAAEAKGPHKLQRQWT  40
                                                         VAATETPEVAAEGDAGAAEAKGPHKLQRQWT
Ta-eIF(iso)4e_T0-1_B1    61 IWEHIV*ISEQF*KFHDLF*NQDFF*KNSVAATETPEVAAEGDAGAAEAKGPHKLQRQWT 120

Ta-eIF(iso)4e_B(WT)      41 FWYDIQTKPKPGAAWGTSLKKGYTFDTVEEFWCLYDQIFRPSKLVGSADFHLFKAGVEPK 100
                            FWYDIQTKPKPGAAWGTSLKKGYTFDTVEEFWCLYDQIFRPSKLVGSADFHLFKAGVEPK
Ta-eIF(iso)4e_T0-1_B1   121 FWYDIQTKPKPGAAWGTSLKKGYTFDTVEEFWCLYDQIFRPSKLVGSADFHLFKAGVEPK 180

Ta-eIF(iso)4e_B(WT)     101 WEDPECANGGKWTVISSRKANLDTMWLETCMALIGEQFDESQEICGVVASVRQRQDKLSL 160
                            WEDPECANGGKWTVISSRKANLDTMWLETCMALIGEQFDESQEICGVVASVRQRQDKLSL
Ta-eIF(iso)4e_T0-1_B1   181 WEDPECANGGKWTVISSRKANLDTMWLETCMALIGEQFDESQEICGVVASVRQRQDKLSL 240

Ta-eIF(iso)4e_B(WT)     161 WTKTASNEAVQVDIGKKWKEVIDYNDKMVYSFHDDSRSQKPSRGGRYTV* 210
                            WTKTASNEAVQVDIGKKWKEVIDYNDKMVYSFHDDSRSQKPSRGGRYTV*
Ta-eIF(iso)4e_T0-1_B1   241 WTKTASNEAVQVDIGKKWKEVIDYNDKMVYSFHDDSRSQKPSRGGRYTV* 290
```

Figure S26

Protein alignment of the *ta-eif(iso)4e* T0 plant 1 B1 allele to the WT.

A

```
Ta-eIF(iso)4e_A(WT)       1 --------MAEVEAALPVAATETPEVAAESDAGAAEAKGPHKLQRQWTFWYDIQTKPKPG  52
                                       +            +    S   A           Q
Ta-eIF(iso)4e_T0-2_A1     1 MALMMSFLRKSICRFSSSGKRR*TDRGTPSRMLATSPAIRDSTNVQNAVSTTSAESFAFF  60

Ta-eIF(iso)4e_A(WT)      53 AAWGTSLKKGYTFDTVEEFWCFFGSLYDQIFRPSKLVGSADFHLFKAGVEPKWEDPECAN 112
                            ++W ++ ++                +   +        +     F             +
Ta-eIF(iso)4e_T0-2_A1    61 SSWASARRRLGHLAQKGLHLRHRRRVLVLV*SDFPSE*AGRKC*FSFIQGWGRTKVGRSR 120

Ta-eIF(iso)4e_A(WT)     113 GGKWTVISSRKANLDTMWLETCMALIGEQFDESQEICGVVASVRQRQDKLSLWTKTASNE 172
                              KW  +                 + G  +   +   G +   R    +         +
Ta-eIF(iso)4e_T0-2_A1   121 VCKWGQMDCDI*QEGQS*YHVA*NVYGSDWRAVR*EPGNLWCCR*CSPETG*AFIMD*DC 180

Ta-eIF(iso)4e_A(WT)     173 AVQVDIGKKWKEVIDYNDKMVYSFHDDSRSQKPSRGGRYTV*--- 214
                                  G  W+E+               R  + S   +
Ta-eIF(iso)4e_T0-2_A1   181 Q**SCSGGHWQEMEGGY*LQ**DGLQLPR*LEKSETKQRWTIHRV 225
```

B

```
Ta-eIF(iso)4e_A(WT)       1 MAEVEAALPVAATETPEVAAESDAGAAEAKGPHKLQRQWTFWYDIQTKPKPGAAWGTSLK  60
                            MAEVEAAL             +       +G              +  P P        K
Ta-eIF(iso)4e_T0-2_A2     1 MAEVEAALRWRRQRPRRSRPRATPVRPRRRGRTSCSGSGPSGTTSRPSPSPAPPGAPRSK  60

Ta-eIF(iso)4e_A(WT)      61 KGYTFDTVEEFWCFFGSLYDQIFRPSKLVGSADFHLFKAGVEPKWEDPECANGGKWTVIS 120
                            +       +           ++     L+    F   + G     +      G
Ta-eIF(iso)4e_T0-2_A2    61 RATPSTPSKSSGACMIRFSVRVSW*EVLI----FIYSRLG*NQSGKIQSVQMGANGL*YL 116

Ta-eIF(iso)4e_A(WT)     121 SRKANLDTMWLETCMALIGEQFDESQEICGVVASVRQRQDKLSLWTKTASNEAVQVDIGK 180
                            + +  L    L+     +      +++   +     + +       +    +  +  + +
Ta-eIF(iso)4e_T0-2_A2   117 AGRPILIPCGLKRVWL*LESSSMRARKFVVLSLVFARDRISFHYGLRLPVMKLFRWTLAR 176

Ta-eIF(iso)4e_A(WT)     181 KWKEVIDYNDKMVYSFHDDSRSQKPSRGGRYTV* 214
                              + ++    +   +       +  +
Ta-eIF(iso)4e_T0-2_A2   177 NGRRLLTTMIRWSTASTMTREVRNQAEVDDTPC- 209
```

Figure S27

Protein alignments of the *ta-eif(iso)4e* T0 plant 2 A1 (A) and A2 (B) alleles to the WT.

A

```
Ta-eIF(iso)4e_B(WT)       1 MAEVEAALPVAATETPEVAAEGDAGAAEAKGPHKLQRQWTFWYDIQTKPKPGAAWGTSLK  60
                            MAEVEAAL                     +G               +          S +
Ta-eIF(iso)4e_T0-2_B1     1 MAEVEAALRWRRQRPRRSRPRATRVRPRRRGRTSCSGSGPSTTSRPSPSPAPPGAPRSKR  60

Ta-eIF(iso)4e_B(WT)      61 KGYTFDTVEEFWCLYDQIFRPSK-LVGSADFHLFKAGVEPKWEDPECANGGKWTVISSRK 119
                               +  +     C+     R S   V    +         K +  +    G    ++ R
Ta-eIF(iso)4e_T0-2_B1    61 ATPSTPSKSSGACMIRFSVRVSW*EVLIFIYSRLG*NQSGKIQSAQMGANGL*-YLAGRP 119

Ta-eIF(iso)4e_B(WT)     120 ANLDTMWLETCMALIGEQFDESQEICGVVASVRQRQDKLSLWTKTASNEAVQVDIGKKWK 179
                              +        + L        + +  ++ S R R        ++ S        G+ W+
Ta-eIF(iso)4e_T0-2_B1   120 ILIPCGLKRVWL*LESSLMKARKFVVLLLVSARDRD*--DCQ*RSCS--------GRHWQ 169

Ta-eIF(iso)4e_B(WT)     180 EVIDYN-DKMVYSFHDDSRSQKPSRGGRYTV*-- 210
                            E+   +  +           +K     R+T+
Ta-eIF(iso)4e_T0-2_B1   170 EMEGGD*LQ**DGLQLPR*LKKSETKQRWTIHGV 203
```

B

```
Ta-eIF(iso)4e_B(WT)       1 MAEVEAALPVAATETPEVAAEGDAGAAEAKGPHKLQRQWTFWYDIQTKPKPGAAWGTSLK  60
                            MAEVEA   V      +  +E     A  K      + W      + +     A G
Ta-eIF(iso)4e_T0-2_B2     1 MAEVEARRRVLVLV*SDFPSE*----AGRKC*FSFIQGWGRTKSGKIQSAQMGANGL*YL  56

Ta-eIF(iso)4e_B(WT)      61 KGYTFDTVEEFWCLYDQIFRPSKLVGSADFHLFKAGVEPKWEDPECANGGKWTVISSRKA 120
                             G                 RP          L   G++  W   E +      ++ +R
Ta-eIF(iso)4e_T0-2_B2    57 AG-----------------RP---------ILIPCGLKRVWL*LESS------LMKAR--  82

Ta-eIF(iso)4e_B(WT)     121 NLDTMWLETCMALIGEQFDESQEICGVVASVRQRQDKLSLWTKTASNEAVQVDIGKKWKE 180
                                + L +    I   +               R   + L+  T +    +
Ta-eIF(iso)4e_T0-2_B2    83 KFVVLLLVSARDRISFHYG-------------LRLPVMKLFR*TLARNGRR*-----LTT 124

Ta-eIF(iso)4e_B(WT)     181 VIDYNDKMVYSFHDDSRSQKPSRGGRYTV* 210
                            +I ++     +   + R+Q      R
Ta-eIF(iso)4e_T0-2_B2   125 MIRWS--TASTMTQEVRNQAEVDDTRC--- 149
```

Figure S28

Protein alignments of the *ta-eif(iso)4e* T0 plant 2 B1 (A) and B2 (B) alleles to the WT.

A

```
Ta-eIF(iso)4e_D(WT)       1 MAEVEAALPVAATETPEVAAEGDAGAAEAKGPHKLQRQWTFWYDIQTKPKPGAAWGTSLK  60
                            MAEVEAAL                     +G              +  P P        K
Ta-eIF(iso)4e_T0-2_D1     1 MAEVEAALRWRRQRPRRSPPRATRVRPRRRGRTSCSGSGPSGTTSRPSPSPAPPGAPRSK  60

Ta-eIF(iso)4e_D(WT)      61 KGYTFDTVEEFWCLYDQIFRPSKLVGSADFHLFKAG--VEPKWEDPECANGGKWTVISSR 118
                            +       +       +            F   + G     K +  +    G    ++ R
Ta-eIF(iso)4e_T0-2_D1    61 RATPSTPSKSSGACMIRFSVRVSW*EVLIFIYSRLG*NQSGKIQSAQMEANGL*-YLAGR 119

Ta-eIF(iso)4e_D(WT)     119 KTNLDTMWLETCMALIGEQFDESQEICGVVASVRQRQDKLSLWTKTASNEAVQVDIGKKW 178
                               +        + L        + +  ++ S R R        +    +  +  + +
Ta-eIF(iso)4e_T0-2_D1   120 PILIPCGLKRVWL*LESSSMKARKFVVLLLVSARDRIS-FHYGLRLPVMKLFRWTLARNG 178

Ta-eIF(iso)4e_D(WT)     179 KEVIDYNDKMVYSFHDDSRSQKPSRGGRYTV* 210
                            + ++    +   +       +  +
Ta-eIF(iso)4e_T0-2_D1   179 RRLLTTMIRWSTASTMTQEVRNQAEVDDTPS- 209
```

B

```
Ta-eIF(iso)4e_D(WT)       1 MAEVEAALPVAATETPEVAAEGDAGAAEAKGPHKLQRQWTFWYDIQTKPKPGAAWGTSLK  60
                                     V  +                           F Y      + G      ++
Ta-eIF(iso)4e_T0-2_D2     1 ----MIRFSVRVSW*---------------------EVLIFIYSRLG*NQSGKIQSAQME  35

Ta-eIF(iso)4e_D(WT)      61 KGYTFDTVEEFWCLYDQIFRPSKLVGSADFHLFKAGVEPKWEDPECANGGKWTVISSRKT 120
                                                   L G     L   G++  W   E +        S +
Ta-eIF(iso)4e_T0-2_D2    36 ANGL*-----------------YLAGRP--ILIPCGLKRVWL*LESS--------SMKAR  68

Ta-eIF(iso)4e_D(WT)     121 NLDTMWLETCMALIGEQFDESQEICGVVASVRQRQDKLSLWTKTASNEAVQVDIGKKWKE 180
                                + L +    I   +            +R    KL  WT   +   +   +  +W
Ta-eIF(iso)4e_T0-2_D2    69 KFVVLLLVSARDRISFHYG-----------LRLPVMKLFRWTLARNGRRLLTTM-IRWST 116

Ta-eIF(iso)4e_D(WT)     181 VIDYNDKMVYSFHDDSRSQKPSRGGRYTV* 210
                                  ++      D
Ta-eIF(iso)4e_T0-2_D2   117 ASTMTQEVRNQAEVDDTPS----------- 135
```

Figure S29
Protein alignments of the *ta-eif(iso)4e* T0 plant 2 D1 (A) and D2 (B) alleles to the WT.
